# Physiologic Transition During Delayed Cord Clamping With Assisted Ventilation in Preterm Infants: A Secondary Analysis of the VentFirst Trial

**DOI:** 10.1001/jamanetworkopen.2025.45258

**Published:** 2025-11-24

**Authors:** Jennifer L. Fang, Karen D. Fairchild, Gina R. Petroni, Marya L. Strand, Jamie B. Warren, Brenda H. Law, Terri E. Gorman, Tina A. Leone, Sumesh P. Thomas, Susan Niermeyer, and the VentFirst Consortium

**Affiliations:** 1 Division of Neonatal Medicine, Department of Pediatric and Adolescent Medicine, Mayo Clinic, Rochester, Minnesota; 2 Division of Neonatology, Department of Pediatrics, University of Virginia, Charlottesville; 3 Division of Translational Research and Applied Statistics, Department of Public Health Sciences, University of Virginia, Charlottesville; 4 Department of Neonatal and Perinatal Medicine, Akron Children’s Hospital, Akron, Ohio; 5 Division of Neonatology, Department of Pediatrics, Oregon Health and Science University, Portland; 6 Division of Neonatology, Department of Pediatrics, University of Alberta, Edmonton, Alberta, Canada; 7 Division of Neonatology, Department of Pediatrics, Brigham and Women’s Hospital, Boston, Massachusetts; 8 Division of Neonatology, Department of Pediatrics, Columbia University, New York, New York; 9 Section of Newborn Critical Care, Department of Pediatrics, University of Calgary, Calgary, Alberta, Canada; 10 Section of Neonatology, Department of Pediatrics, University of Colorado, Aurora

## Abstract

**Importance:**

Assisted ventilation during delayed cord clamping (DCC) may improve the physiologic transition of extremely preterm infants immediately after birth.

**Objective:**

To determine whether assisted ventilation during 120 seconds of DCC was associated with reduced higher-level resuscitative interventions (intubation, chest compressions, or epinephrine administration) compared with DCC for 30 to 60 seconds followed by resuscitation.

**Design, Setting, and Participants:**

This was a secondary analysis of the VentFirst randomized clinical trial that was conducted from September 2, 2016, through February 21, 2023, at 12 centers in the US and Canada. Infants born at 23 weeks 0 days’ to 28 weeks 6 days’ gestational age (GA) were included.

**Intervention:**

Infants randomized to the intervention received either positive-pressure ventilation or continuous positive airway pressure from 30 to 120 seconds after birth, followed by umbilical cord clamping. Those randomized to control received 30 to 60 seconds of DCC followed by assisted ventilation.

**Main Outcomes and Measures:**

The main outcome was the odds of higher-level resuscitative interventions in the delivery room (DR). Intention-to-treat analyses within 2 a priori cohorts (infants breathing well and not breathing well 30 seconds after birth) used the Cochran-Mantel-Haenszel method to estimate the odds ratios (ORs) of intervention vs control.

**Results:**

All 570 infants enrolled in the trial were included. Infants had a median (IQR) GA of 26.6 (25.2-27.9) weeks and 273 (47.9%) were female. A total of 271 infants (47.5%) were assessed as not breathing well 30 seconds after birth (150 intervention and 121 control), and 299 (52.5%) were assessed as breathing well 30 seconds after birth (128 intervention and 171 control). In the not-breathing-well cohort, 146 infants (53.9%) were intubated in the DR, 4 received chest compressions (2 intervention and 2 control), and 1 received epinephrine (control). Intubation was less frequent in the intervention group (71 infants [47.3%] vs 75 infants [62.0%]; OR, 0.52; 95% CI, 0.30-0.89). When adjusted by GA strata at randomization, infants in the 26 to 28 weeks’ GA stratum who were in the intervention group were less likely to be intubated in the DR (18 of 79 infants [22.8%] vs 29 of 60 infants [48.3%]; OR, 0.32; 95% CI, 0.15-0.65). However, there was no difference in intubation rates for infants in the 23 to 25 weeks’ GA stratum (53 of 71 infants [74.7%] vs 46 of 61 infants [75.4%]; OR, 0.96; 95% CI, 0.44-2.12). Among infants breathing well at 30 seconds, 74 (24.7%) were intubated in the DR, and none received compressions or epinephrine. Intubation rates were similar between intervention and control in the breathing-well cohort.

**Conclusions and Relevance:**

While the VentFirst trial did not find a difference in death or intraventricular hemorrhage, this secondary analysis found that assisted ventilation during DCC was associated with less intubation in the DR, primarily among infants born at 26 to 28 weeks’ gestation who were not breathing well 30 seconds after birth. Additional studies are needed before implementing assisted ventilation during DCC in clinical practice.

**Trial Registration:**

ClinicalTrials.gov Identifier: NCT02742454

## Key Points

**Question:**

Is assisted ventilation during delayed cord clamping (DCC) associated with diffrences in physiologic transition after birth in extremely preterm infants?

**Findings:**

In this secondary analysis including 570 infants from the VentFirst randomized clinical trial of infants born at less than 29 weeks’ gestation, assisted ventilation during DCC was associated with significantly reduced odds of intubation in the delivery room for infants not breathing well 30 seconds after birth. For infants breathing well at 30 seconds, assisted ventilation during DCC was not associated with rates of intubation in the delivery room.

**Meaning:**

These findings suggest that assisted ventilation during DCC may improve physiologic transition, particularly for infants with respiratory depression.

## Teaser

This secondary analysis of the VentFirst trial compares varying levels of assisted ventilation and delayed cord clamping (DCC) and their associations with higher-level resuscitative interventions and physiologic transition among infants born preterm.

## Introduction

Delayed umbilical cord clamping (DCC) compared with immediate or early cord clamping reduces mortality for preterm infants,^1,2,3^ with a recent individual participant data meta-analysis^2^ demonstrating more than a 30% reduction in odds of death prior to hospital discharge. In addition to survival benefits, DCC may lead to a more stable transition from fetal to neonatal circulation,^4^ as evidenced by its association with higher Apgar scores.^1,2^

Many trials of DCC have excluded preterm infants who were not breathing well after birth, limiting generalizability of these trials to clinical practice. Experimental physiology studies in preterm lambs suggest that assisted ventilation during DCC results in a smoother cardiovascular and pulmonary transition.^5,6^ This includes higher right ventricular output, more stable heart rate, greater pulmonary blood flow, and more stable cerebral blood flow and pressure. The impact of assisted ventilation during DCC on the cardiorespiratory transition and resuscitative interventions in extremely preterm infants remains unclear.

The primary objective of this secondary analysis of data from the VentFirst randomized clinical trial^7^ was to evaluate whether assisted ventilation during 120 seconds of DCC was associated with reduced higher-level resuscitative interventions (intubation, chest compressions, or epinephrine administration) compared with DCC for 30 to 60 seconds followed by assisted ventilation. In the VentFirst trial we analyzed 2 a priori cohorts separately: infants breathing well and those not breathing well 30 seconds after birth and infants randomized according to gestational age (GA) strata (23 to 25 and 26 to 28 weeks). We hypothesized that the association of assisted ventilation during DCC with higher-level resuscitative interventions would differ based on breathing cohort and GA stratum. Secondary outcomes included markers of physiologic transition in the delivery room (DR), including heart rate, Apgar scores, infant temperature, volume expansion, and surfactant administration.

## Methods

This is a secondary analysis of the VentFirst trial and was was deemed exempt by the University of Virginia institutional review board due to the use of deidentified data. Reporting follows the Consolidated Standards of Reporting Trials (CONSORT) reporting guideline. In the VentFirst trial, conducted from September 2, 2016, through February 21, 2023, at 12 study sites throughout the US and Canada, pregnant women expected to deliver at 23 weeks 0 days’ to 28 weeks 6 days’ gestation who provided written informed consent to participate were randomized shortly before delivery to their infants receiving assisted ventilation from 30 to 120 seconds after birth followed by cord clamping (intervention) or DCC for 30 to 60 seconds after birth followed by assisted ventilation (control). Analyses were based on intention-to-treat within 2 a priori cohorts: infants breathing well and those not breathing well 30 seconds after birth. The trial protocol is provided in (Supplement 1). A full description of study methods can be found in the primary trial publication.^7^ The institutional review board of each center approved the protocol.

The prespecified outcomes of this secondary study included the following resuscitative interventions and DR measures: intubation, chest compressions, epinephrine administration, volume bolus, surfactant administration, heart rate at 1 minute after birth, Apgar scores at 1 and 5 minutes, and infant temperature in the DR. Given the pragmatic (ie, delivery of the intervention in a usual care setting) trial design, indications for intubation and surfactant administration in the DR were not specified by study protocol; sites followed their unit-based clinical criteria for these interventions. Indications to start chest compressions or administer epinephrine in the DR were per neonatal resuscitation program guidelines. When infant temperature in the DR was not recorded, Neonatal Intensive Care Unit admission temperature was used. Heart rate was derived from the 1-minute Apgar score as absent, less than 100 beats per minute (bpm), or greater than 100 bpm.

### Statistical Analysis

Outcomes were examined within cohort, both with and without adjustment for GA at randomization. The Cochran-Mantel-Haenszel test was used to estimate odds ratios (ORs) and 95% CIs by study intervention, both unadjusted and adjusted according to a 2-level GA stratification factor.

Given the underlying goal of assessing associations with DR measures and resuscitative interventions, logistic regression analyses used actual GA at the time of delivery, not GA at the time of randomization, and categorizations of time of cord clamping instead of group at randomization. Seventeen infants were in the lower GA stratum at randomization but in the higher GA stratum at delivery. For these analyses, GA at birth (in weeks) was adjusted as a continuous measure, and cord clamping time (in seconds) was adjusted as an ordinal 5-level categorization (group 1, <15 seconds; group 2, 15-29 seconds; group 3, 30-59 seconds; group 4, 60-119 seconds; group 5, ≥120 seconds). Due to the observed site variability in the primary analysis, it was deemed important to include adjustment for site as a class variable in the models. Of the 12 study sites, 4 enrolled fewer than 50 infants and were grouped as one, leading to an 8-level site factor. Backward and stepwise regression strategies were used to determine which factors, when added to the model that included GA, cord clamping time, and site, were associated with outcomes. Graphical displays, scatterplots, and other diagnostics, including review of variance inflation factors and tolerance, were used to assess model assumptions, correlations, and multicollinearity. All reported results have variance inflation factors of less than 4 and tolerance greater than 0.25. Model fitting assessments included Pearson and deviance residuals, leverage, and predicted probability diagnostics. All analyses were carried out using SAS 9.4 (SAS Institute). Statistical significance was defined as a 2-sided *P* < .05.

## Results

All 570 infants enrolled in the VentFirst trial, including 22 sets of twins, were included in this secondary analysis (eFigure 1 in Supplement 2). Three infants were excluded from analysis of DR temperature because of missing data, and time of cord clamping was missing for 1 infant. Infants had a median (IQR) GA of 26.6 (25.2-27.9) weeks, including 273 females (47.9%) and 297 males (52.1%) (Table 1). All mothers received at least 1 dose of antenatal steroids, with 91.8% (523 of 570 mothers) receiving 2 or more doses. A total of 271 infants (47.5%) were assessed as not breathing well 30 seconds after birth (150 intervention and 121 control), and 299 (52.5%) were assessed as breathing well 30 seconds after birth (128 intervention and 171 control). Infants not breathing well at 30 seconds were more likely to be male, of lower GA and birth weight, and delivered by cesarean delivery compared with infants assessed as breathing well at 30 seconds.

**Table 1.** Infant Characteristics by Randomization Before Birth

Characteristic	Participants, No. (%)					
	Total		Not breathing well at 30 s		Breathing well at 30 s	
	Intervention (n = 278)	Control (n = 292)	Intervention (n = 150)	Control (n = 121)	Intervention (n = 128)	Control (n = 171)
GA stratum, wk^a^						
23 wk 0 d to 25 wk 6 d	116 (41.7)	115 (39.4)	71 (47.3)	61 (50.4)	45 (35.2)	54 (31.6)
26 0 d to 28 wk 6 d	162 (58.3)	177 (60.6)	79 (52.7)	60 (49.6)	83 (64.8)	117 (68.4)
GA at birth, median (IQR), wk	26.6 (24.9-27.7)	26.6 (25.4-27.9)	26.0 (24.4-27.6)	26.1 (24.7-27.4)	26.9 (25.6-28.0)	27 (25.9-28.0)
Birth weight, median (IQR), g	840 (650-1030)	813 (658-988)	760 (600-970)	750 (575-920)	885 (728-1065)	850 (708-1028)
Sex						
Female	132 (47.5)	141 (48.3)	65 (43.3)	54 (44.6)	67 (52.3)	87 (50.9)
Male	146 (52.5)	151 (51.7)	85 (56.7)	67 (55.4)	61 (47.7)	84 (49.1)
Twin gestation	16 (5.8)	28 (9.6)	9 (6.0)	9 (7.4)	7 (5.5)	19 (11.1)
Preeclampsia	98 (35.3)	108 (37.0)	54 (36.0)	45 (37.2)	44 (34.4)	63 (36.8)
Preterm prelabor rupture of membranes	102 (36.7)	111 (38.0)	56 (37.3)	48 (39.7)	46 (35.9)	63 (36.8)
Chorioamnionitis, clinical	48 (17.3)	47 (16.1)	27 (18.0)	21 (17.4)	21 (16.4)	26 (15.2)
Antenatal steroids						
1 Dose	21 (7.6)	26 (8.9)	14 (9.3)	11 (9.1)	7 (5.5)	15 (8.8)
≥2 Doses	257 (92.5)	266 (91.1)	136 (90.7)	110 (90.9)	121 (94.5)	156 (91.2)
Magnesium day of delivery	258 (92.8)	254 (87.0)	137 (91.3)	101 (83.5)	121 (94.5)	153 (89.5)
Cesarean delivery	186 (66.9)	207 (70.9)	111 (74.0)	98 (81.0)	75 (58.6)	109 (63.7)

Abbreviation: GA, gestational age.

^a^ GA at randomization.

**Figure 1.**
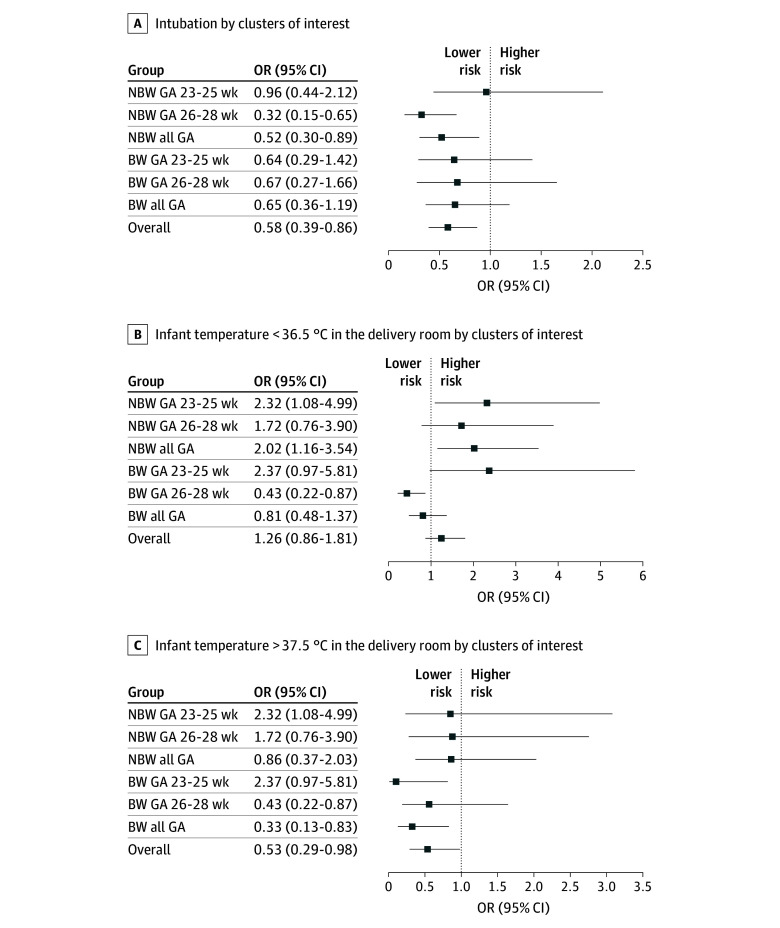
Odds of Intubation, Hypothermia, and Hyperthermia in the Delivery Room Hypothermia was defined as less than 36.5 °C, and hyperthermia was defined as greater than 37.5 °C. Gestational age (GA) is stratified by weeks. BW indicates breathing well; NBW, not breathing well; OR, odds ratio.

Evaluation of the primary outcome (ie, higher-level resuscitative interventions) in the not-breathing-well cohort showed that 53.9% of infants (146 infants) were intubated in the DR; four infants received chest compressions (2 intervention and 2 control), and 1 infant received epinephrine (1 control) (Table 2). Intubation was less frequent in the intervention group compared with the control group (71 infants [47.3%] vs 75 infants [62.0%]; OR, 0.52; 95% CI, 0.30-0.89) (Figure 1A). When odds of intubation were adjusted for GA strata, infants in the 26 to 28 weeks’ GA stratum who received the intervention were less likely to require intubation (18 of 79 infants [22.8%] vs 29 of 60 infants [48.3%]; OR, 0.32; 95% CI, 0.15-0.65) (Figure 1A and eTable 1 in Supplement 2). However, for infants in the 23 to 25 weeks’ GA stratum, there was no detectable difference in intubation rates between intervention and control (53 of 71 infants [74.7%] vs 46 of 61 infants [75.4%]; OR, 0.96; 95% CI, 0.44-2.12) (Figure 1A and eTable 1 in Supplement 2). The number of infants who received chest compressions or epinephrine in the DR was too low to allow for between-group comparison.

**Table 2.** Resuscitative Interventions and Indicators of Physiologic Transition by Randomization Before Birth, Adjusted for GA Stratum at Randomization

Intervention or indicator	Not breathing well at 30 s				Breathing well at 30 s			
	Participants, No. (%)		OR (95% CI)	MD (95% CI)	Participants, No. (%)		OR (95% CI)	**MD (95% CI)**
	Intervention (n = 150)	Control (n = 121)			Intervention, (n = 128)	Control (n = 171)		
Primary outcome in the delivery room								
Intubation (all)	71 (47.3)	75 (62.0)	0.52 (0.30 to 0.89)	NA	28 (21.9)	46 (26.9)	0.65 (0.36 to 1.19)	NA
Chest compressions	2 (1.3)	2 (1.7)	Too few to analyze	NA	0	0	NA	NA
Epinephrine administration	0	1 (0.8)	Too few to analyze	NA	0	0	NA	NA
Other measures in the delivery room								
Time of cord clamping, median (IQR), s^a^	105 (20-122)	30 (5-35)	NA	NA	120 (119-125)	62 (60-66)	NA	NA
Umbilical artery pH, median (IQR)	7.2 (7.1-7.3)	7.3 (7.2-7.3)	NA	−0.02 (−0.05 to 0.01)	7.3 (7.2-7.3)	7.3 (7.2-7.3)	NA	0.001 (−0.02 to 0.03)
Apgar score at 1 min, median (IQR)	3 (2-5)	2 (1-3)	NA	0.98 (0.56 to 1.40)	6 (5-7)	6 (5-7)	NA	0.02 (−0.38 to 0.42)
Apgar score at 5 min, median (IQR)	7 (5-8)	6 (5-7)	NA	0.23 (−0.23 to 0.69)	8 (7-8)	8 (7-8)	NA	−0.03 (−0.35 to 0.28)
Heart rate >100 bpm at 1 min after birth	63 (42.0)	29 (24.0)	2.30 (1.35 to 3.88)	NA	100 (78.1)	132 (77.2)	1.07 (0.62 to 1.86)	NA
Volume bolus administration	8 (5.3)	12 (9.9)	0.52 (0.20 to 1.31)	NA	3 (2.3)	4 (2.3)	0.96 (0.21 to 4.41)	NA
Surfactant administration	49 (32.7)	60 (49.6)	0.52 (0.26 to 1.02)	NA	19 (14.8)	38 (22.2)	0.90 (0.55 to 1.46)	NA
Infant temperature, median (IQR), °C^b^	36.6 (36.3-36.9)	36.8 (36.5-37.0)	NA	−0.02 (−0.05 to 0.01)	36.7 (36.5-37.1)	36.8 (36.4-37.1)	NA	0.001 (−0.02 to 0.03)
Infant temperature <36.5 °C^b^	51 (34.6)	25 (20.8)	2.02 (1.16 to 3.54)	NA	31 (24.2)	48 (28.2)	0.81 (0.48 to 1.37)	NA
Infant temperature >37.5 °C^b^	6 (4.0)	9 (7.5)	0.86 (0.37 to 2.03)	NA	6 (4.7)	19 (11.2)	0.33 (0.13 to 0.84)	NA

Abbreviations: bpm, beats per minute; GA, gestational age; MD, mean difference; NA, not applicable; OR, odds ratio.

^a^ Cord clamping time was not available for 1 infant in the intervention group in the not breathing well cohort.

^b^ Three infants (0.5%) had no recorded temperature, and 38 infant temperatures (6.5%) in the delivery room were not available. Neonatal intensive care unit admission temperature was used.

When evaluating the primary outcome among the breathing-well cohort, 24.7% of infants (74 infants) were intubated in the DR, and no infants received chest compressions or epinephrine (Table 2). Intubation rates were similar between intervention and control groups (28 infants [21.9%] vs 46 infants [26.9%]; OR, 0.65; 95% CI, 0.36-1.19) (Figure 1A). There was no detectable difference in intubation rates between intervention and control in either GA stratum for infants who were breathing well (23-25 weeks’ GA stratum: 20 of 45 infants [44.4%] vs 30 of 54 infants [55.6%]; OR, 0.64; 95% CI, 0.29-1.42; 26-28 weeks’ GA stratum: 8 of 83 infants [9.6%] vs 16 of 117 infants [13.7%]; OR, 0.67; 95% CI, 0.27-1.66) (Figure 1A and eTable 1 in Supplement 2).

To further understand the risk factors for intubation in the DR, we performed logistic regression modeling adjusted for study design factors (breathing cohort, cord clamping time, GA, and site). The model demonstrated that in addition to these factors, maternal preeclampsia (OR, 0.29; 95% CI, 0.18-0.48) and heart rate greater than 100 bpm 1 minute after birth (OR, 0.41; 95% CI, 0.25-0.67) were negatively associated with DR intubation. Delay in cord clamping of 120 seconds or greater, as compared with clamping at less than 15 seconds, was associated with reduced likelihood of intubation (OR, 0.45; 95% CI, 0.22-0.95) (Table 3). Model statistics are provided in eTable 2 in Supplement 2. Additional infant characteristics used in logistic regression models shown in eTable 3 in Supplement 2.

**Table 3.** Models for Intubation, Hypothermia (< 36.5 °C), and Hyperthermia (>37.5 °C) in the Delivery Room

Variable	Regression coefficient (SE)	Wald χ^2 ^(*df*)	*P* value	OR (95% Wald CI)
Logistic regression model results for intubation^a^				
BW^b^	−0.728 (0.278)	6.853 (1)	.009	0.48 (0.28-0.83)
GA at birth, wk	−0.682 (0.082)	69.528 (1)	<.001	0.51 (0.43-0.59)
DR CC , s^c^	NA	6.173 (4)	.19	NA
DR CC group 5 vs 1^b^^,^^d^	−0.542 (0.235)	5.339 (1)	.02	0.45 (0.22-0.95)
DR CC group 4 vs 1^b^^,^^d^	0.012 (0.230)	0.003 (1)	.96	0.79 (0.38-1.63)
DR CC group 3 vs 1^b^^,^^d^	0.140 (0.265)	0.278 (1)	.60	0.90 (0.41-1.98)
DR CC group 2 vs 1^b^^,^^d^	0.143 (0.428)	0.112 (1)	.74	0.90 (0.29-2.84)
Site^c^	NA	45.191 (7)	<.001	NA
Apgar heart rate at 1 min >100 bpm^b^	−0.890 (0.253)	12.386 (1)	<.001	0.41 (0.25-0.67)
Maternal preeclampsia^b^	−1.239 (0.252)	24.153 (1)	<.001	0.29 (0.18-0.48)
Logistic regression model results for hypothermia^e^				
BW^b^	−0.002 (0.245)	0.000 (1)	>.99	1.00 (0.62-1.61)
GA birth, wk	−0.008 (0.085)	0.008 (1)	.93	0.99 (0.84-1.17)
DR CC, s^c^	NA	1.543 (4)	.82	NA
DR CC group 5 vs 1^b^^,^^d^	0.106 (0.216)	0.243 (1)	.62	1.23 (0.62-2.44)
DR CC group 4 vs 1^a^^,^^d^	0.229 (0.211)	1.175 (1)	.28	1.39 (0.71-2.72)
DR CC group 3 vs 1^b^^,^^d^	0.106 (0.257)	0.171 (1)	.68	1.23 (0.57-2.62)
DR CC group 2 vs 1^b^^,^^d^	−0.343 (0.434)	0.626 (1)	.43	0.78 (0.25-2.49)
Site^c^	NA	49.455 (7)	<.001	NA
Birth weight, kg	−1.827 (0.593)	9.494 (1)	.002	0.16 (0.05-0.51)
Maternal cesarean delivery^b^	0.539 (0.256)	4.414 (1)	.04	1.71 (1.04-2.83)
Logistic regression model results for hyperthermia^f^				
BW^b^	−0.005 (0.343)	0.000 (1)	.99	1.00 (0.51-1.95)
GA birth, wk	−0.090 (0.127)	0.510 (1)	.48	0.91 (0.71-1.17)
DR CC, s^c^	NA	5.213 (4)	.27	NA
DR CC group 5 vs 1^b^^,^^d^	0.071 (0.395)	0.032 (1)	.86	0.44 (0.18-1.11)
DR CC group 4 vs 1^b^^,^^d^	0.366 (0.382)	0.916 (1)	.34	0.60 (0.25-1.44)
DR CC group 3 vs 1^b^^,^^d^	0.154 (0.440)	0.123 (1)	.73	0.48 (0.17-1.34)
DR CC group 2 vs 1^b^^,^^d^	−1.474 (1.148)	1.649 (1)	.20	0.10 (0.01-1.67)
Site^c^	NA	10.206 (7)	.18	NA
Birth weight, kg	1.775 (0.802)	4.894 (1)	.03	5.90 (1.22-28.42)
Maternal clinical chorioamnionitis^b^	0.833 (0.344)	5.848 (1)	.02	2.30 (1.17-4.52)

Abbreviations: bpm, beats per minute; BW, breathing well; ; CC, cord clamped; DR, delivery roomGA, gestational age; OR, odds ratio.

^a^ Model includes 569 infants, of whom 219 (38.0%) were intubated. One infant was missing CC time.

^b^ Indicator variable.

^c^ Categorical variable.

^d^ Group 1, less than 15 seconds; group 2, 15 to 29 seconds; group 3, 30 to 59 seconds; group 4, 60 to 119 seconds; group 5, 120 seconds or greater.

^e^ Model includes 566 infants of whom 154 (27.0%) were hypothermic. One infant was missing CC time, and 3 infants were missing DR temperature.

^f^ Model includes 566 infants, of whom 51 (9.0%) were hyperthermic. One infant was missing CC time, and 3 infants were missing DR temperature.

Examining other markers of physiologic transition in the not-breathing-well cohort revealed notable differences between intervention and control groups (Table 2). Infants who were not breathing well 30 seconds after birth and randomized to the intervention had higher 1-minute Apgar scores (median [IQR], 3 [2-5] vs 2 [1-3]; mean difference, 0.98 points; 95% CI, 0.56-1.40 points) and were more likely to have a heart rate greater than 100 bpm 1 minute after birth (63 of 150 infants [42.0%] vs 29 of 121 infants [24.0%]; OR, 2.30; 95% CI, 1.35-3.88). There was no between-group difference in surfactant administration or receipt of a volume bolus in the DR. For infants who were breathing well 30 seconds after birth, there were no detectable differences in other markers of physiologic transition between intervention and control, including Apgar scores, heart rates, and surfactant or volume bolus administration (Table 2).

Thermoregulation is an important aspect of physiologic transition at birth and differed in infants randomized to the intervention compared with controls. Infants not breathing well 30 seconds after birth randomized to the intervention were more likely to be hypothermic in the DR (temperature <36.5 °C; 51 infants [34.6%] vs 25 infants [20.8%]; OR, 2.02; 95% CI, 1.16-3.54) (Figure 1B). Infants in the 23 to 25 weeks’ GA stratum were especially vulnerable (29 of 71 infants [41.4%] vs 14 of 61 infants [23.3%]; OR, 2.32; 95% CI 1.08-4.99) (eTable 1 in Supplement 2). When evaluating risk of hyperthermia (infant temperature >37.5 °C), there was no significant between-group difference in odds of hyperthermia for infants who were not breathing well (Figure 1C). However, for infants who were assessed as breathing well 30 seconds after birth, hyperthermia was less likely for those who received the intervention (6 infants [4.7%] vs 19 infants [11.2%]; OR, 0.33; 95% CI, 0.13-0.84) (Table 2). This was particularly true for infants in the 23 to 25 weeks’ GA stratum (1 of 45 infants [2.2%] vs 10 of 54 infants [18.5%]; OR, 0.10; 95% CI, 0.01-0.82) (eTable 1 in Supplement 2).

**Figure 2.**
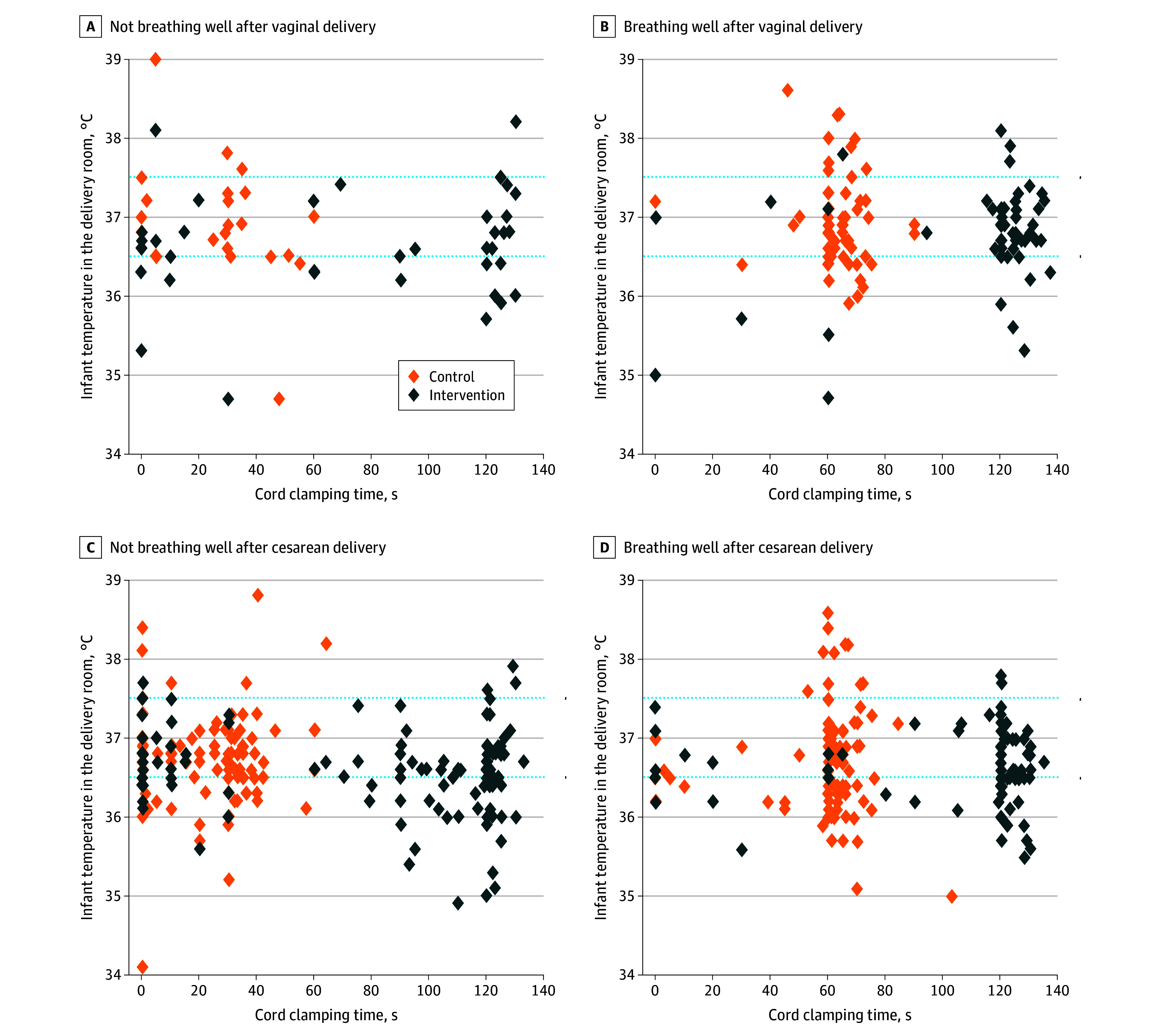
Individual Infant Temperatures in the Delivery Room by Actual Cord Clamping Time, Mode of Delivery, Cohort, and Group Delivery modes were vaginal vs cesarean delivery. The cohort included those breathing well vs not breathing well 30 seconds after birth in both the intervention (assisted ventilation during 120 seconds of delayed cord clamping) and control (delayed cord clamping for 30 to 60 seconds followed by assisted ventilation) groups.

Given the apparent association of study group with thermoregulation, we further assessed both hypothermia and hyperthermia in the DR by cohort, birth weight, mode of delivery, and actual cord clamping time. Cord clamping time did not appear to be associated with hypothermia or hyperthermia in the DR in either cohort regardless of whether the infant was delivered vaginally or via cesarean delivery (Figure 2). In both study groups and both breathing cohorts, lower birth weight was associated with infant hypothermia in the DR, especially for infants born via cesarean delivery (eFigure 2 in Supplement 2). Logistic regression models for infant temperature in the DR (Table 3) showed hypothermia was associated with birthweight (OR, 0.16; 95% CI, 0.05-0.51), cesarean delivery (OR, 1.71; 95% CI, 1.04-2.83), and study site. Hyperthermia was associated with birthweight (OR, 5.90; 95% CI, 1.22-28.42) and maternal chorioamnionitis (OR, 2.30, 95% CI, 1.17-4.52). Model statistics are provided in eTable 2 in Supplement 2, and additional infant characteristics used in logistic regression models are shown in eTable 3 in Supplement 2.

## Discussion

In this secondary analysis of the VentFirst trial evaluating the physiologic transition after delivery, infants not breathing well 30 seconds after birth who received assisted ventilation during DCC were less likely to require intubation in the DR. This finding was primarily observed in infants born at 26 to 28 weeks’ gestation. In addition, assisted ventilation during DCC for infants not breathing well was associated with improved transition after birth as demonstrated by higher 1-minute Apgar scores and greater likelihood of heart rate greater than 100 bpm at 60 seconds. The intervention was also associated with more frequent hypothermia and less hyperthermia in the DR.

Our findings of a favorable association of assisted ventilation during DCC with the physiologic transition after birth for extremely preterm infants not breathing well differ from those of 2 earlier randomized clinical trials.^8,9^ In a study of assisted ventilation during 60 seconds of DCC compared with DCC alone, Katheria et al^8^ did not detect any differences in DR interventions, including intubation and surfactant administration, nor improvement in other metrics such as Apgar scores, heart rate during the first 10 minutes after birth, or infant temperature in the DR. The ABC New Zealand study,^9^ a single-center trial of preterm infants less than 30 weeks’ gestation not breathing well 15 seconds after birth, compared positive pressure ventilation with gentle stimulation during 50 seconds of DCC. This study^9^ found no significant difference in intubation, chest compressions, epinephrine, Apgar scores, hypothermia, or surfactant administration in the DR. Compared with these trials,^8,9^ the VentFirst trial had a longer duration of assisted ventilation during DCC, included infants of lower GA, and had a much larger sample size.

An even larger multicenter trial with longer duration of DCC was the Netherlands Aeration, Breathing, Clamping Study 3 (ABC3),^10^ which compared physiological-based with time-based cord clamping in infants less than 30 weeks’ gestation. Infants in the intervention group received ventilation during DCC until they achieved cardiorespiratory stability (median cord clamping time of 5 minutes and 47 seconds), while infants in the control group had their cord clamped after 30 to 60 seconds (median cord clamping time of 47 seconds). In that trial,^10^ the primary outcome (death, severe brain injury, or necrotizing enterocolitis) was not significantly different overall, but male infants experienced higher intact survival. Intubation in the DR was much lower than in the VentFirst trial, and higher-level resuscitative interventions did not differ between intervention and control groups. The ABC3 trial^10^ did not distinguish between infants breathing well or not breathing well prior to the intervention. Importantly, an individual participant data meta-analysis of randomized trials of assisted ventilation during DCC is planned by the iCOMP group.^11^

The lower rate of DR intubation in the not-breathing-well cohort that received the intervention in the VentFirst trial may be due to improved lung aeration and pulmonary blood flow while infants received oxygenated blood from the placenta.^12^ Using continuous positive airway pressure or positive pressure ventilation to establish functional residual capacity prior to separation from the placental circulation may have allowed more infants to continue noninvasive respiratory support in the DR. Additionally, preclinical models have shown that providing breathing support during DCC increases pulmonary venous return to the left atrium, thereby stabilizing cardiac preload after the umbilical cord is clamped.^13^ More stable hemodynamics may explain the higher Apgar scores and heart rates at 1 minute with the VentFirst intervention.

When modeling intubation in the DR, we found maternal preeclampsia, higher GA, breathing-well status at 30 seconds, heart rate greater than 100 bpm at 1 minute, and DCC of 120 seconds or greater were associated with decreased odds of DR intubation. Less intubation with higher GA is expected^14,15^ given the greater lung maturity and surfactant production occurring with increasing GA. Similarly, independent breathing effort 30 seconds after birth and good heart rate are reassuring signs of stability—making intubation less likely. We speculate that lower DR intubation rates for infants born to mothers with preeclampsia may be attributable to accelerated fetal lung maturity with chronic stress,^16,17^ more optimally timed antenatal steroids, or lower risk of intraamniotic infection, which is associated with inflammation-mediated respiratory depression.^18,19^ We also found that hospital site was associated with odds of intubation in the DR, which aligns with other studies demonstrating that DR practices vary between hospitals.^20,21^

While assisted ventilation during DCC may facilitate the cardiorespiratory transition for preterm infants not breathing well after delivery, efforts are needed to maintain normothermia. Extremely preterm infants require thermoregulatory interventions in the DR,^13^ and DCC has been associated with increased risk of hypothermia on Neonatal Intensive Care Unit admission.^2,10^ While plastic wrap and exothermic warming mattresses were used during the VentFirst intervention, infants who were not breathing well had an increased incidence of hypothermia in the DR. Hypothermia was more common in the lower GA stratum and for those delivered by cesarean delivery. Study site also influenced the risk of hypothermia, which may be due to practice variation or differences in experience with extremely preterm deliveries. These results highlight the importance of having standard processes for thermal protection and monitoring infant temperature in the DR, with the necessary environmental controls, equipment, and team training to avoid both hypothermia and hyperthermia; this is especially important as practice moves toward longer delays in cord clamping for all preterm infants, in light of increasing evidence of feasibility and benefits.^11^

### Limitations

The VentFirst trial and this secondary study have many strengths, including the large accrual number across multiple sites in North America as well as the pragmatic study design, but there are also limitations. While infants in the intervention group received assisted ventilation during DCC, we have limited information on the effectiveness of the breathing support, because not all infants had capnography and none had respiratory function monitoring before cord clamping. Also, it is possible that clinicians, who could not be blinded to study group, were biased in assignment of Apgar scores and decisions about intubation, indications for which were not standardized in the protocol. Infant heart rate at 1 minute was not objectively measured but instead derived from the 1-minute Apgar score. Additionally, because antenatal consent and time for equipment set-up were required, the trial excluded the sickest infants who required urgent or emergent delivery, limiting the generalizability of the study findings. Future studies should focus on whether and how providing ventilatory support prior to cord clamping influences physiologic transition after birth and impacts important clinical outcomes for extremely preterm infants. This includes questions related to the optimal duration of resuscitation with intact cord, type of breathing support and amount of supplemental oxygen delivered based on the infant’s clinical status, and the thermal interventions needed to prevent hypo- and hyperthermia.

## Conclusions

While the VentFirst trial did not detect a difference in death or intraventricular hemorrhage, this secondary analysis found assisted ventilation during 120 seconds of DCC was associated with reduced odds of intubation in the DR for infants not breathing well 30 seconds after birth, primarily for those born at 26 to 28 weeks’ gestation. The intervention was also associated with improved markers of cardiopulmonary stability for infants not breathing well initially. Attention to thermoregulation is important because the intervention was associated with lower infant temperature in the DR.

## Notes

Supplement 1. Trial Protocol

Supplement 2. eFigure 1. CONSORT Diagram of VentFirst Participants eTable 1. Primary Outcome and Delivery Room Measures With Potential Association Within Gestational Age Strata at Randomization eTable 2. Model Statistics eTable 3. Additional Infant Outcome Characteristics Used in Logistic Regression Models eFigure 2. Individual Infant Temperatures in the Delivery Room by Birth Weight, Mode Of Delivery (Vaginal Versus Cesarean Section), Cohort (Breathing Well Versus Not Breathing Well 30 seconds after birth), and Arm (Intervention Versus Control)

Supplement 3. Nonauthor Collaborators

Supplement 4. Data Sharing Statement
