# Supplementary material for: Physiologic Transition During Delayed Cord Clamping With Assisted Ventilation in Preterm Infants: A Secondary Analysis of the VentFirst Trial
Source: JAMA Netw Open. 2025 Nov 24;8(11):e2545258. doi: 10.1001/jamanetworkopen.2025.45258 (PMC12645335; doi:10.1001/jamanetworkopen.2025.45258)
Supplement: Supplement 1. — Trial Protocol [file jamanetwopen-e2545258-s001.pdf]

**Study Title**	**VentFirst:** A Multicenter RCT of Assisted Ventilation During Delayed Cord Clamping for Extremely Preterm Infants
**Study Intervention**	Providing assisted ventilation prior to umbilical cord clamping for extremely preterm infants to decrease risk of intraventricular hemorrhage
**Indication Studied**	Birth of an extremely preterm baby
**Sponsor**	NICHD
**Sponsor Protocol Number**	NICHD: 1R01HD087413-01<br>UVA IRB #:18783
**Development Phase of Study**	Phase III
**Release Date**	Protocol Version 6.0 August 29, 2023
**GCP Statement**	This study is to be performed in full compliance with acceptable Good Clinical Practices (GCP) as required by U.S. Code of Federal Regulations applicable to clinical studies (45CFR46), ICH GCP E6 and completion of Human Subjects Protection Training. All required study documentation will be archived as required by regulatory authorities.

Principal Investigator:

Karen Fairchild, MD

Scientific Advisor: John

Kattwinkel,

MD

Participating Institutions:

University of Virginia	Charlottesville, VA
Brigham & Women's Hospital	Boston, MA
Mayo Clinic	Rochester, MN
St. Louis University	St Louis, MO
University of Colorado	Aurora, CO
Oregon Health & Science University	Portland, OR
University of Calgary	Calgary, Alberta
Columbia University	New York, NY
Indiana University	Indianapolis, IN
University of California, Davis	Sacramento, CA
University of Alberta, Edmonton	Edmonton, Alberta
University of Alabama at Birmingham	Birmingham, AL

Biostatistician: Gina Petroni, PhD

SIGNATURE PAGE

Signature:		
Name:	Karen Fairchild, MD<br>Role: PI	Date

INVESTIGATOR'S AGREEMENT

I confirm that I have read this protocol and I agree to conduct the study as outlined herein. I agree to conduct the study in accordance with the ethical principles that have their origin in the Declaration of Helsinki and that are consistent with Good Clinical Practices as outlined in ICH E6 and the applicable laws and regulations.

Site Investigator:	Signature	Date
	Name	

Instructions to the Site Investigator: Please sign and date this signature page. File the original signature page in the study file at the site and send a copy of the signed and dated page to the UVA Study PI or designee.

STUDY DESIGN

This is a multicenter, randomized clinical trial (RCT) of 30-60 second (Standard arm) versus 120 seconds (VentFirst arm) delayed umbilical cord clamping for Extremely Preterm (EPT) infants with ventilatory assistance given either after umbilical cord clamping in the Standard arm or prior to umbilical cord clamping in the VentFirst arm.

- No additional interventions or testing are involved.
- Routine clinical, laboratory, and head ultrasound imaging data will be collected from the medical record until 36 weeks' postmenstrual age.
- The primary outcome is intraventricular hemorrhage by 7-10 days after birth. Secondary outcomes are listed below.
- A Data Safety and Monitoring Committee will oversee the study.

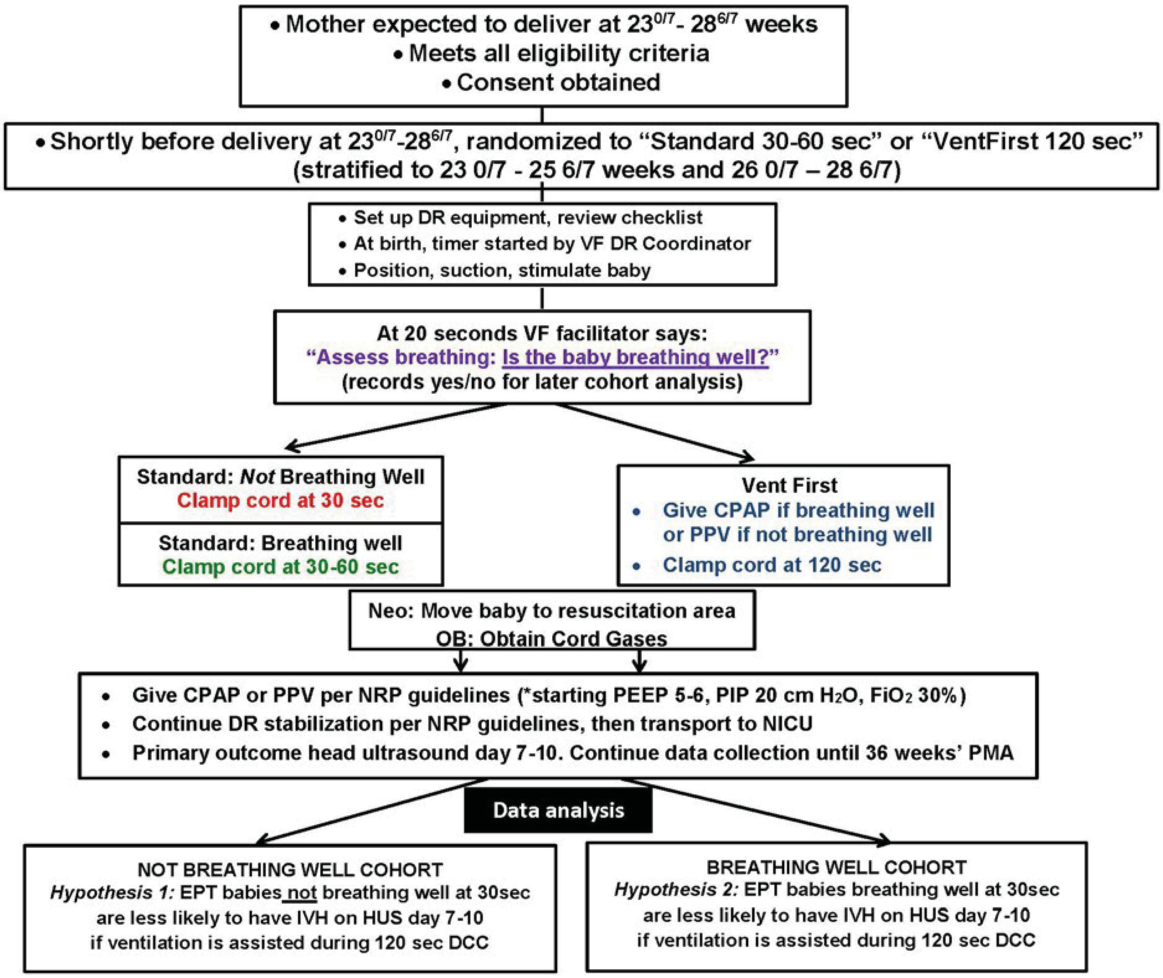

SYNOPSIS

**Title of Study: VentFirst: A Multicenter Randomized Clinical Trial of Assisted Ventilation During Delayed Cord Clamping for Extremely Preterm Infants**	
**Investigator(s): Karen Fairchild, MD**	
**Study centers:** Twelve sites in the United States and Canada.	
**Phase of study: III**	**Planned Study Initiation:** April, 2016
**Study Duration:** 5 years	**Length of Subject Participation:** Study intervention occurs in the first 2 minutes after birth. Study data are collected from birth until 36 weeks postmenstrual age.

Objectives: To test the following hypotheses

- Extremely preterm infants (23 $^{0/7}$ to 28 $^{6/7}$ weeks' gestational age) not breathing well by 30 seconds from birth are less likely to have IVH on head ultrasound 7-10 days from birth if they receive assisted ventilation from 30 to 120 seconds followed by cord clamping, compared to those with cords clamped at 30-60 sec, followed by assisted ventilation.
- Extremely preterm infants (23 $^{0/7}$ to 28 $^{6/7}$ weeks' gestational age) breathing well by 30 seconds from birth are less likely to have IVH on head ultrasound 7-10 days from birth if they receive assisted ventilation from 30 to 120 seconds followed by cord clamping, compared to those with cords clamped at 30-60 sec, followed by assisted ventilation.

Outcomes:
Primary:

- IVH (any grade) on head ultrasound 7-10 days after birth

Secondary: Incidence of adverse events related to the following:

- Immediate post-delivery interventions
- Hematologic and cardiovascular parameters during the first 24 hours after birth.
- Hematologic and respiratory interventions in the first 10 days after birth
- Complications and conditions related to prematurity though 36 weeks post-menstrual age.

Methodology: Randomized clinical trial of delivery room intervention for extremely preterm infants. Intervention cannot be blinded but primary outcome will be assigned by expert radiologists at non-study institutions who will be blinded to study arm.

Total Number of infants planned: 940

Study population: Extremely preterm infants

Inclusion criteria:

- 23 $^{0/7}$ – 28 $^{6/7}$ weeks gestation at delivery
- Written informed consent obtained before delivery

Exclusion criteria:

- Life-threatening condition of fetus (including but not limited to: severe hydrops, lethal chromosomal abnormality, severe congenital malformation)
- Suspected severe fetal anemia
- Monochorionic twins
- Multiple gestation greater than twins
- Decision made for comfort care only
- Medical emergency necessitating emergency delivery (e.g., complete placental abruption)
- Obstetrician or Neonatology concern for inappropriateness of the study intervention based on maternal or fetal factors (must specify reason in screening log)

Investigational intervention: Assisted ventilation during delayed cord clamping. Infants randomized to the **Standard Arm** (delayed cord clamping at 30-60 seconds) will have assisted ventilation after cord clamping. Infants randomized to the **VentFirst Arm** (delayed cord clamping at 120 seconds) will have assisted ventilation from 30-120 sec after birth, prior to cord clamping.

Other study intervention(s): None

Criteria for evaluation:

Efficacy: Head ultrasound imaging 7-10 days after birth and other clinical outcomes

Safety: Incidence and severity of Serious Unanticipated Events, SAEs and AEs determined to be related or possibly related to the intervention.

Statistical methods: The study is designed as a Phase III, two-arm, randomized, multicenter comparative study of **Standard** versus **VentFirst** cord clamping in extremely preterm infants in two cohorts of infants (those **breathing well** and those **not breathing well**). Analyses will be performed separately within each cohort.

Efficacy: The primary analysis will be based on all randomized eligible EPT infants, and will be based on the arm they were randomized to regardless of intervention received (intent-to-treat). The primary outcome of the trial is based upon the central review of the head ultrasound and is the proportion of EPT infants who are alive without any grade IVH on head ultrasound obtained 7-10 days following birth. The primary outcome classification is binary and the stratified Cochran-Mantel-Haenszel test for relative risk will be used for the final comparison of intervention arms.

Safety: All randomized EPT infants will be evaluable for adverse events from the time of delivery. Adverse events will be tabulated by frequency and severity, overall and by arm as well as by cohort. Differences in the level of adverse events by study intervention will be assessed by classifying them as severe or not severe and examining the relative proportion of severe events.

LIST OF ABBREVIATIONS

**Abbreviation or specialist term**	**Explanation**
AE	Adverse Event
ACOG	American College of Obstetrics and Gynecology
AAP	American Academy of Pediatrics
BPD	Bronchopulmonary Dysplasia
CBC/Diff	Complete Blood Count and differential
CPAP	Continuous Positive Airway Pressure
CFR	Code Of Federal Regulations
CI	Confidence Interval
CRC	Clinical Research Coordinator
CRF	Case Report Form
DCC	Delayed Cord Clamping
DR	Delivery Room
DSMC	Data and Safety Monitoring Committee
EPT	Extremely Preterm (< 29 weeks gestation)
GA	Gestational Age
GCP	Good Clinical Practices
HUS	Head ultrasound
ICH	International Conference On Harmonization
IRB	Institutional Review Board<br>(or equivalent board in Canada)
IVH	Intraventricular Hemorrhage
MOP	Manual of Procedures
MuSIC Database	Multi-Schema Information Capture
NICU	Neonatal Intensive Care Unit
NRP	Neonatal Resuscitation Program
PPV	Positive Pressure Ventilation
SAE	Serious Adverse Event
TI	(Randomization) Transaction Identifier
USAE	Unanticipated Serious Adverse Event
UVA	University of Virginia
UVA CTO	UVA Clinical Trials Office
VFF	VentFirst Facilitator

Table of Contents

1 BACKGROUND AND RATIONALE 2
2 STUDY OBJECTIVES 6
3 SUBJECT ELIGIBILITY 7
4 TREATMENT PLAN 8
5 EVALUATIONS AND ASSESSMENTS 16
6 OUTCOME MEASURES 18
7 STATISTICAL CONSIDERATIONS 22
8 ADVERSE EVENTS AND REPORTING 30
9 DATA SAFETY MONITORING PLAN 35
10 STUDY MANAGEMENT 36
11 REFERENCES 39

1 BACKGROUND AND RATIONALE

Study Synopsis

VentFirst is a phase III multicenter randomized clinical trial of assisted ventilation during delayed cord clamping (DCC) for extremely preterm infants, (EPT, born at <29 weeks' gestation). The study is sponsored by the National Institute of Child Health and Human Development and aims to enroll 940 EPT infants over a 5-year period at 10 NICUs across the US and Canada. The primary goal of VentFirst is to determine whether providing ventilatory assistance prior to umbilical cord clamping reduces the incidence of intraventricular hemorrhage (IVH), compared to standard practice which is cord clamping at 30-60 seconds after birth, with ventilatory assistance provided afterwards. Secondary outcomes include reduction in other preterm morbidities.

Disease Background

IVH is a significant problem for infants born EPT and can result in neurodevelopmental disabilities.[1] IVH is thought to be due in part to shifts in cerebral blood flow and blood pressure, some of which may occur immediately after birth.[2] Facilitating a smooth cardiorespiratory transition at birth is an important goal of neonatology resuscitation teams. In contrast to infants born at term, many EPT infants do not breathe well on their own after birth. In the past, standard practice had been to cut the umbilical cord of EPT newborns immediately after birth and transfer of the infant to the neonatology team who could provide assisted ventilation. Recent evidence indicates that delaying umbilical cord clamping for 30 or more seconds improves outcomes of preterm infants.[3] Even more recently, animal studies have shown that achieving lung inflation prior to cord clamping results in a smoother cardiovascular transition, [4] which might reduce IVH risk in preterm infants with fragile brain microvasculature.

Study Intervention Rationale

The rationale for VentFirst can be summarized as follows (with details provided below):

1) Delayed cord clamping has been shown to be beneficial in preterm infants that are spontaneously breathing
2) If infants are not breathing well, most providers feel it is necessary to clamp the cord early so as not to delay resuscitation by the neonatology team.

3) Many EPT infants do not breathe well, so are not able to benefit from DCC
4) If assisted ventilation were provided during DCC, more EPT infants could benefit from the extra time attached to the placenta.
5) Potentially even more importantly, assisting ventilation during DCC may lead to a smoother cardiovascular transition. Preclinical studies have shown that achieving lung inflation prior to cord clamping stabilizes the blood pressure, heart rate, and cerebral blood flow and oxygenation, compared to cord clamping before lung inflation. If this is the case in humans, then IVH risk could be lower with assisted ventilation prior to cord clamping.

Rationale for Delayed Cord Clamping (DCC): Clinical Studies

Multiple small randomized clinical trials of DCC for preterm infants have been published, showing some advantages and low risk. In a meta-analysis of 14 studies of delayed (30-180 sec) compared to immediate/early (~15 sec) clamping for preterm infants, advantages of DCC included a higher hematocrit, less need for blood transfusion, less need for vasopressor administration, and a lower incidence of intraventricular hemorrhage (IVH) and necrotizing enterocolitis.[3]

With regard to disadvantages, it was previously thought that DCC might lead to hypothermia, polycythemia, hyperbilirubinemia, and delayed resuscitation. However, hypothermia and polycythemia were not found to be significant problems in randomized clinical trials, and bilirubin was higher and duration of phototherapy longer in some but not all studies.
Whether DCC resulted in delayed resuscitation could not be assessed in these studies because in most cases if a baby was judged to require resuscitation the team disregarded the study assignment and clamped the cord early.[5]

Rationale for Assisted Ventilation prior to Cord Clamping: Preclinical Studies

Many EPT infants do not breathe well on their own immediately after birth, and their umbilical cord is clamped before their lungs are inflated. This puts them at a significant disadvantage physiologically. When the umbilical cord is cut and the placental circulation removed before the

lungs are inflated, the following adverse transition occurs: 1) blood return from placenta to right atrium (a major component of cardiac preload) abruptly ceases 2) pulmonary vascular resistance remains elevated and blood bypasses the fluid-filled lungs shunting right to left through the ductus arteriosus 3) the under filled left ventricle pumps against high systemic vascular resistance (rather than to the low resistance placenta), compromising cardiac output.[6]

Recent evidence in preterm animal models suggests that inflating the lungs prior to cord clamping is beneficial. Lambs delivered at the equivalent of 28 weeks of human gestation either had the umbilical cord clamped immediately and assisted ventilation provided after, or had assisted ventilation prior to cord clamping. Lambs in the “Clamp First” group had significant decline in heart rate and oxygenation and swings in cerebral blood flow compared to lambs in the “Vent First” group that had more stable cardiovascular and cerebral blood flow parameters.[6] In extremely preterm infants, rapid changes in cerebral blood pressure and flow if the cord is cut before the lungs are inflated may lead to ischemic injury or intraventricular hemorrhage. Since many EPT infants do not breathe well on their own immediately after birth, the goal of the VentFirst RCT is to test whether providing ventilatory assistance during DCC reduces IVH and other common morbidities of EPT infants.

Rationale for Study Design

VentFirst is a randomized clinical trial of a delivery room intervention for EPT infants with the goal of reducing IVH, a major complication of EPT birth. Infants randomized to the Standard Arm will receive current standard practice for EPT infants, as recommended by ACOG in 2012,[7] of 30-60 sec DCC. Cord clamping at 30 sec if not breathing well and 60 sec if breathing well has been suggested; however, given recent shifts in clinical practice, it is at the discretion of the OB and Neonatology teams to delay cord clamping past 30 seconds, but not longer than 60 seconds, even if the baby is not breathing well.[14] Standard Arm infants will receive assistance with ventilation after the cord is clamped. Infants randomized to the VentFirst Arm will receive assisted ventilation from 30-120 sec after birth, while attached by the umbilical cord to the placenta. The cord will be clamped at 120 sec after birth. Both groups will get standard breathing assistance and other resuscitation measures, the only difference being timing of cord clamping and timing and location of beginning assisted ventilation. In both groups, initial assisted ventilation will be either CPAP (continuous positive

airway pressure) via face mask if the EPT infant is breathing or PPV (positive pressure ventilation) via face mask if the infant is not breathing well. Endotracheal intubation will be performed as per standard NRP guidelines if the infant is not responding to PPV via face mask. Per protocol, intubation will not be performed until after cord clamping (although the lead neonatologist present may elect to clamp the cord early, should intubation be felt to be emergently indicated before the 120-second delay prescribed by protocol).

The study was designed primarily to determine whether EPT infants not breathing well on their own will benefit from assisted ventilation during prolonged placental transfusion. Infants breathing well on their own might also benefit from assisted ventilation and extra time attached to the placenta before cord clamping, but we expect this effect to be smaller.

Of note, infants randomized to the “Standard” group will be getting 30-60 seconds of DCC, which is within the range of previous clinical trials and is the current recommendation from ACOG. It is not known whether 120 sec of DCC is more beneficial than 30-60 sec. This would not be feasible to study for this population, since most practitioners would not accept delaying resuscitation of non-breathing EPT infants for 120 seconds during DCC.

The study intervention cannot be blinded but the primary outcome will be assigned by expert radiologists at non-study institutions who will be blinded to study arm.

2 STUDY OBJECTIVES

2.1 Hypotheses

1) Extremely preterm infants (EPT, 23 $^{0/7}$ to 28 $^{6/7}$ weeks' gestational age) **not breathing well** by 30 seconds from birth are less likely to have IVH on head ultrasound 7-10 days from birth if they receive assisted ventilation from 30 to 120 seconds followed by cord clamping, compared to those with cords clamped at 30-60 seconds, followed by assisted ventilation.

2) EPT infants **breathing well** by 30 seconds from birth are less likely to have IVH on head ultrasound 7-10 days from birth if they receive assisted ventilation from 30 to 120 seconds followed by cord clamping, compared to those with cords clamped at 30-60 seconds, followed by assisted ventilation.

2.2 Specific Aim

Conduct a randomized clinical trial to test the above two hypotheses to facilitate development of the following guidelines for resuscitation of EPT infants at birth:

If both hypotheses are supported: All EPT infants should have DCC for 120 seconds, with assisted ventilation provided prior to cord clamping.

If hypothesis 1 is supported, but hypothesis 2 is not: All EPT infants should have DCC for at least 30 seconds, and if the infant is *not breathing* by 30 seconds, assisted ventilation should be provided while the cord remains un- clamped until 120 seconds after birth.

If both hypotheses are refuted: All EPT infants should have DCC for at least 30 seconds (based on previous studies), but there is insufficient evidence to recommend more prolonged DCC with assisted ventilation for those not breathing well at 30 seconds.

3 SUBJECT ELIGIBILITY

Infants born at participating sites who fulfill the inclusion and exclusion criteria listed below are eligible for enrollment in this study. Mothers of all races and ethnicities will be approached for consent. Maternal written informed consent will be obtained prior to delivery.

Inclusion Criteria (All inclusion criteria MUST be present)

[1] $23^{0/7}$ – $28^{6/7}$ weeks gestation at delivery

[2] Written informed consent obtained before delivery

Exclusion Criteria (All exclusion criteria MUST NOT be present)

[1] Life threatening condition of fetus (including but not limited to: severe hydrops, lethal chromosomal abnormality, severe congenital malformation)

[2] Suspected severe fetal anemia

[3] Monochorionic twins

[4] Multiple gestation greater than twins

[5] Decision made for comfort care only

[6] Medical emergency necessitating emergency delivery (e.g., complete placental abruption)

[7] Obstetrician or Neonatology concerns for inappropriateness of the study intervention based on other maternal or fetal factors (must specify reason on the screening log)

Twins

Centers may consider enrolling twins once they have completed the VentFirst intervention on at least 3 singleton C-section Vent First deliveries, in order to gain experience with setting up and carrying out the VentFirst protocol. At that point, the site PI and site Obstetrician can opt to begin approaching **mothers of twins (dichorionic only)** about participation in the study. Team training on logistics of twin VentFirst deliveries should be carried out prior to randomizing any twin gestations. The site PI or site Obstetrician may opt to not enroll twins.

4 TREATMENT PLAN

Study Overview

This is a multicenter, randomized, phase III clinical trial that will randomize 940 EPT infants into one of two study arms, stratified by gestational age ($23^{0/7} – 25^{6/7}$ weeks and $26^{0/7} - 28^{6/7}$ weeks):

1) **Standard Arm** with delayed cord clamping at 30-60 seconds and assisted ventilation provided after cord clamping

2) **VentFirst Arm** with delayed cord clamping at 120 seconds and assisted ventilation provided before cord clamping.

The study intervention will occur in the first 2 minutes after birth. Initial assisted ventilation in both groups will consist of application of continuous positive airway pressure (CPAP) or positive pressure ventilation (PPV) via face mask. Study data will be collected in the delivery room and from the infant's medical record from birth until 36 weeks postmenstrual age (PMA = gestational age at birth + age from birth) or until discharge home, whichever comes first. Maternal data will be collected from the mother's chart until her hospital discharge after delivery. If an infant is transferred to an outside facility prior to 36 weeks PMA, a member of the study team will follow-up with the outside facility regarding outcomes to 36 weeks PMA. Permission to contact the outside facility will be obtained from the mother prior to conducting this follow-up.

The study is being conducted at 12 sites in the United States and Canada. It is expected that accrual will take place over 5 years.

Screening

At each site, medical records of women hospitalized and expected to deliver at $23^{0/7}- 28^{6/7}$ weeks gestation will be reviewed by a member of the study team for study eligibility.

After medical record review, if *all* of the inclusion criteria and *none* of the exclusion criteria are met, the mother will be approached by a member of the study team to provide consent for herself and her infant to participate in the study. A study consent video might be available to help explain the study. The video would be approved by each sites' IRB and would NOT replace the written informed consent process.

There may be occasions where a mother signs consent and then is discharged home prior to delivery**.** At readmission, a member of the study

team must confirm that all study inclusion criteria are *met* and all exclusion criteria *not met* prior to randomization.

Enrollment

Once informed consent is obtained, the mother-infant dyad is considered enrolled in the study. The following registration data will be entered into the study database (MuSIC, Multi-Schema Information Capture):

- Registering site and investigator
- Date of signed informed consent
- Mother's demographic data
- Estimated gestational age of the fetus
- Documentation of study eligibility

Detailed instructions for the registration process can be found in the study Manual of Procedures (MOP).

Randomization

Study eligibility and consent must be confirmed prior to randomization.

Randomization should be done as close to the expected time of delivery as possible, preferably within one hour. At each site, randomization will be done by the Clinical Research Coordinator (CRC) or a trained member of the study team via a web-based platform.

The CRC or trained team member will login to the randomization module, enter the current estimated gestational age, and request a study arm assignment. The server will provide the arm assignment based on a randomization scheme specified by the project biostatistician along with a single use alphanumeric transaction identifier consisting of a color and a 2-digit number, such as "red51." The server will display the transaction identifier, site code, transaction time, gestational age and arm assignment to the requesting personnel.

A delivery room script specific to the study arm "Standard 30-60 second" or "VentFirst-120 second" will be available to the neonatology team attending the delivery. The transaction identifier must be recorded on the appropriate delivery script.

Twins will automatically be assigned to the same study intervention and will have the same transaction identifier but will be assigned a unique subject number in the MuSIC database.

Delivery Room Study Intervention

VentFirst Team Roles

VentFirst deliveries will be attended by NRP-trained neonatology providers who usually attend EPT births. This typically includes some combination of a neonatology attending physician, neonatology fellow physician, and/or Neonatal Nurse Practitioner. Providers will be trained in the VentFirst protocol using computer-based learning modules, training videos, and simulation sessions with regular updates as specified in the MOP.

For infants randomized to the VentFirst arm, a trained and NRP-certified neonatology provider will be assigned to carry out the 2-minute intervention after birth.

A VentFirst Facilitator (VFF) will be assigned after randomization and before delivery. This individual will have a clipboard with a script and a timer and will be responsible for timing the interventions and reading the protocol script from birth to 120 seconds. The VFF will also write DR data on the VF Script. An audio recorder may also be used for quality control to confirm timing of interventions. The site CRC and PI may use the audio recordings to review the data written on the DR script by the VFF. Audio recordings will be identified by subject ID, stored in a secure location at each site for quality control review, and made available to the UVA study team during site audits if needed.

Delivery Preparation

- If randomization is to the VentFirst Arm and delivery is by Cesarean section, a neonatology provider will be sterilely scrubbed and dressed to enter the operating field. Equipment needed for assisting ventilation that might contact the sterile field will be sterilized or placed in a sterile wrap, or will be single use removed from company packaging immediately before delivery when sterilization is not feasible. The Fisher & Paykel company (F&P) has agreed to provide a gas-sterilized version of their standard masks and tubing for delivering CPAP and/or positive pressure ventilation to study subjects who are being delivered by Cesarean section. However, since the sterile packaging is not yet commercially available, F&P will package the standard sterilized equipment in specially-labeled packaging and require that the equipment be used for VentFirst study subjects only. VentFirst sites can opt to use the sterilized masks provided by (F&P) if they choose.

- Equipment for providing CPAP and PPV, oxygen and air tanks and blender, and flowmeter which are standard for resuscitation of EPT infants will be on a mobile stand for deliveries randomized to VentFirst resuscitation prior to cord clamping. See the study MOP for VentFirst equipment configuration requirements and instructions.
- A VFF will be assigned to record time of birth and will read the VentFirst delivery script which gives instructions for the respective arm and will record basic delivery room data on the DR script (See MOP for the delivery script). The VFF will record the number of seconds after birth that the cord was clamped and, if the time is more than 15 seconds shorter or longer than the assigned time, will record the reason for this variance. An audio recorder may be activated prior to delivery and the audio tape may be reviewed by the CRC or site PI after delivery to verify data written on the DR script.

At Delivery

Initial Steps (both arms)

- For **vaginal** deliveries, the infant will be placed on a stable surface near the introitus on a warming pad with OB towel and/or plastic drape to prevent hypothermia.
- For **Cesarean section** deliveries, the infant will be placed on the mother's pelvis or a suitable stable surface on a sterilely covered warming pad and sterile OB towel and/or sterile plastic drape.
- A heart rate monitor may be applied.
- The infant will be stimulated to breathe for up to 30 seconds from birth, including bulb suctioning of the mouth and nose if indicated.
- At 20 seconds from birth, the VFF will announce "20 seconds; Neo assess breathing. Is the baby breathing well or not?" The neonatology provider will assess breathing, and at 30 seconds announce "breathing well", or "not breathing well" and the VFF will record this.

Standard Arm

- If the infant is randomized to the Standard 30-60 sec arm, the cord can be clamped at **30 sec if the infant is *NOT* breathing well.** The option to delay cord clamping, past 30 seconds but NOT longer than 60 seconds, if the infant is NOT breathing well is at the discretion of the OB and Neonatology teams. This option is consistent with the current ACOG

guideline of allowing 30-60 seconds of DCC for preterm infants. **If the infant is breathing well, the cord is clamped at 60 sec.** Heart rate will be checked at 50 seconds prior to cord clamping at 60 seconds. The infant is then moved to the resuscitation area for ventilatory assistance, either CPAP if breathing well or PPV if not, per NRP guidelines (see below).

- If the infant is designated as breathing well at 30 seconds but stops breathing prior to 60 seconds, the cord may be clamped prior to 60 seconds at the discretion of the OB and Neonatology providers.
- Although some obstetricians and neonatologists have advocated that the umbilical cord be "milked", as an alternative to delayed cord clamping, umbilical cord milking is not an approved part of the intervention in either standard or VentFirst arms. If the cord is milked prior to clamping, this will be noted on the DR Script and would be considered a protocol deviation. Umbilical cord milking is not recommended as a standard practice in national and international guidelines[8,9].

VentFirst Arm

- At 30 seconds, the neonatology provider will give CPAP if the infant is breathing well or PPV if not. Ventilatory assistance will continue from 30 seconds until the script reader announces "120 seconds, clamp cord".
- If, during the intervention, an infant getting CPAP stops breathing, PPV will start. Conversely, if an infant is getting PPV and starts breathing, CPAP will be given.
- For infants in the VentFirst arm, per NRP guidelines, heart rate will be assessed after CPAP or PPV are initiated. This will be done either by brief gentle cord palpation or with a heart rate monitor if available. HR will be assessed at 60 and 90 seconds. If at either time the HR is <100 beats/minute, standard NRP steps will be done to improve ventilation and HR. This includes repositioning the mask, suctioning the mouth/nose, opening the mouth slightly, or increasing the pressure of the PPV breaths. If HR is persistently low, at the discretion of the OB and Neonatal providers the cord may be clamped prior to 120 seconds.

NOTE: *Settings for ventilatory assistance for both groups will be based on standard NRP guidelines[8] and each hospital's protocols, with FiO_2 30%, PEEP 5-6 cm H_2O, Peak Inspiratory Pressure 20 cm H_2O (with option to increase if the heart rate is low or PPV is judged to be inadequate).*

Post-Delivery Instructions and Data Collection (both arms)

Infant (neonatology team):

- Number of seconds after birth that the cord was actually clamped is recorded by the VFF. For quality control purposes, audio recording of the DR intervention may be performed for later review by the site CRC or PI to validate data written on DR script.
- If the cord was clamped more than +/- 15 seconds from the target time required in the specific study arm (30-60, or 120 sec), the VFF will record a reason on the delivery data sheet and a protocol deviation will be reported (see section 10.2.2).
- After the baby is moved to the resuscitation table, further resuscitation measures are carried out per NRP guidelines.[8]

Mother (OB team):

- If oxytocin is given to the mother after delivery, the guideline will be togive after cord clamping as noted below.
- Umbilical cord arterial and venous blood gases will be requested per routine care after the cord is cut.
- If the mother has acute post-partum hemorrhage (≥1000 ml estimated blood loss within 24 hours of delivery), this will be noted as an SAE as described below and in the MOP. If the mother has a retained placenta, this will also be recorded in the database and reported as an AE. Post-partum delivery-related infection prior to the mother's hospital discharge will also be recorded and reported as an AE. These data will be sought in the mother's medical record.

NOTE: In case of an unanticipated emergency, the Obstetrician or Neonatologist may terminate or deviate from the protocol.

Prohibited and Concomitant Medications

All medications, interventions, procedures and blood products normally provided as part of routine care are permitted in this study. Data collected for the purposes of the study will be limited to the administration of the specific medications or blood products listed below. This data will be entered into the CRF's by the CRC based on review of the mother's and infant's medical records.

Data collected on maternal medications prior to and after delivery:

- o Number of prenatal steroid doses
- o Magnesium given within 24 hours prior to delivery
- o Oxytocin given after delivery (and timing related to cord clamping)

NOTE: It will be recommended that if oxytocin is given to the mother during the third stage of labor (after delivery of the infant and before delivery of the placenta) *it should be given after the cord is clamped.*

Data collected on infant medications:

- o Number of surfactant doses from birth to 10 days
- o Volume boluses during the first 24 hours after birth
- o Medications for low blood pressure during the first 24 hours after birth
- o Indomethacin given in the first 2 days after birth for IVH prevention
- o Medication given after day 2 to treat a patent ductus arteriosus
- o Total number of red blood cell transfusions given from birth to 10 days

Duration of Study Participation

The study intervention will take place in the first 2 minutes after birth.

Follow-up will be done from birth to 36 weeks PMA or until hospital discharge (whichever comes first) and then again at 18 to 24 months corrected gestational age (CGA). The 18 – 24 month follow-up will involve a Neurodevelopmental clinic visit done for routine care.

Discontinuation/Withdrawal from Study

Participants may be withdrawn or be discontinued from the study at any time for any of the following reasons:

- Mother withdraws consent
- Obstetrician or neonatology physician determines that a maternal or fetal condition precludes the study intervention
- Sponsor PI closes the study
- The DSMC or a local IRB deems it necessary

If a mother withdraws consent prior to birth, the study intervention will not be performed and the mother’s prenatal data and the reason for withdrawal of consent with be documented in the study database.

If maternal consent is withdrawn at any point after the study intervention, all data obtained prior to the time of consent withdrawal will be used for data analysis, but data will not be collected after withdrawal.

All participants that withdraw from the study will receive standard treatment.

Subject Status Definitions

Enrolled: A mother-infant dyad is considered “enrolled” in the study once the mother has signed the consent form.

Randomized: A mother-infant dyad is considered “randomized” at the time a study intervention assignment is allocated from the randomization module.

On-Study: A mother-infant dyad is considered “on-study” from the time of randomization, unless consent is withdrawn.

On Follow-up: A mother-infant dyad is considered “on follow-up” once the study intervention is completed.

Off-Study: The mother is considered “off study” at her discharge from the hospital after delivery. The infant is considered “off-study” once the infant reaches 36 weeks PMA, is discharged to home or dies, whichever comes first.

5 EVALUATIONS AND ASSESSMENTS

Time and Events Table

Table 1: SCHEDULE OF EVENTS

Assessments / Procedures	Pre-Study	Day 1 (Birth Day)	Follow-Up 1st 24h	HUS at 7-10 days	Follow- Up 1st 10 days	Final HUS 36 wks PMA +/- 7days	Follow-up 36 wks PMA[5] +/-_ 7 days	Follow-Up 18-24 months CGA
Review inclusion/exclusion criteria	X							
Obtain Informed Consent	X							
Medical history	X							
Confirm eligibility[1]		X						
Randomization		X						
Vent First Study intervention		X						
Data collection + labs[2]		X	X				X	
Head ultrasound (HUS)[3]				X		X	X	
Adverse events reporting[4]		X	X	X	X	X	X	
Bayley III exam[6]								X
ASQ questionnaire[6]								X
MCHAT-R questionnaire[6]								X
General health assessment								X

1. Eligibility *must* be confirmed prior to study intervention. This is particularly important for mothers who have consented and are discharged and readmitted.
2. VentFirst delivery data collection will be done in real time in the delivery room. See the Vent First delivery forms. See other CRF's for all other follow-up data collection required.
3. A window of up to 14 days post birth is allowed to repeat the day 7-10 HUS if the original scan was judged to be inadequate by site radiology attending.
4. See Section 8 for description of expedited vs routine reporting requirements.
5. If an infant is discharged to another facility prior to 36 wks PMA final follow-up, a member of the study team will obtain maternal consent and then will contact the outside facility to obtain status at 36 weeks PMA.
6. Bayley III exam, ASQ and MCHAT are standard neurodevelopmental tests routinely performed on preterm infants at 18-24 months CGA. .

Pre-Study Intervention Assessments

There are no study-specific assessments required prior to the VentFirst intervention. All procedures, assessments, and laboratory tests prior to delivery are those that would be done as part of routine care.

Study Assessments

There are no specific on-study assessments required for this protocol other than that the mother-infant dyad must meet all eligibility criteria prior to consent *and* prior to randomization.

Follow-up Assessments

After the delivery room intervention, infants are followed for clinical outcomes and adverse events until 36 weeks PMA or until death or discharge home, whichever comes first. A 36 week PMA chart review follow-up will be done by a member of the study team. If the infant has been transferred to an outside facility prior to 36 weeks PMA a study team member will obtain maternal consent and follow-up with the outside facility to collect the relevant study data. At 18 – 24 months CGA infants will return for a Neurodevelopmental follow-up clinic visit that is part of routine care. Neurodevelopmental follow-up data, such as results from Bayley III testing, ASQ and MCHAT-R questionnaires and general health data will be collected for study purposes. General health information can include data such as: current medications, issues with chronic lung disease, seizures, and surgeries or hospitalizations after NICU discharge.

Data Collection

The UVA Multi-Schema Information Capture (MuSIC) database will be used by all sites for electronic data entry. Delivery room data will be written on the DR script by the VFF or Neonatology provider during or immediately following the intervention. Some of the data may be available in the electronic medical record and/or on audiotape for validation. The data will be entered into the MuSIC database by the clinical research coordinator (CRC) after delivery.

With the exception of some of the study intervention delivery data, all data required for this study should be documented in the infant's medical record as part of routine care.

All study data should be entered into MuSIC by each site based on the data entry guidelines specified in the MOP.

6 OUTCOME MEASURES

All outcomes measures will be part of routine care and recorded in the medical record, with the exception of outside expert readings of HUS at 7-10 days and last HUS at birth hospital prior to 36 weeks.

Specification of the Appropriate Outcome Measures

Multiple head ultrasounds are performed as routine care for all EPT infants. At each site, HUS will be performed 7-10 days after birth (nearly all IVH occurs before this time), and this will be used for primary outcome assessment. Failure to obtain a 7-10 day HUS will be considered as a protocol deviation, and the subsequent HUS accepted for the purposes of determining the primary outcome as discussed in section 7 on statistics.

The HUS performed at the birth hospital closest to the 36 week PMA follow-up time point will be used for the secondary HUS outcome assessment. The primary reason for the late HUS is to evaluate for cystic periventricular leukomalacia, which is usually not present on the 7-10 day HUS but can be associated with adverse perinatal and birth events and is associated with adverse long-term neurologic outcomes. The final HUS is also important for assessing post-hemorrhagic hydrocephalus, which evolves over time and is associated with severe (Grade III-IV) IVH and adverse long-term neurologic outcomes. If a center performs brain MRI rather than HUS near 36 weeks PMA, this will be used for secondary outcomes assessment.

For clinical care and for adverse event reporting, all HUS's will be read per standard of care by board-certified pediatric radiologists trained in neonatal HUS interpretation at each study site. These reports will not be used for primary or secondary study outcomes assessment.

For study endpoints, an independent radiologist at non-study institutions (blinded to study assignment) will independently read the 7-10 day and the final 36 week PMA HUS on each study infant. These readings will be performed in batch at intervals to be determined by the statistical analysis plan and DSMC requirements. Any HUS with discrepant findings between the local and the independent radiologist that would impact primary outcome assessment will be evaluated by another independent radiologist.

Quality review of each HUS will be performed by board-certified pediatric radiologists trained in neonatal HUS interpretation at each study site. If

studies are judged to be of poor quality for standard interpretation, they will be repeated as part of routine clinical care.

Detailed instructions regarding transmitting images to the independent radiologists, details of the HUS central reading center process, and HUS quality assurance can be found in the study MOP.

For the purposes of study endpoints assessment, head ultrasounds will be interpreted based on standard criteria of Papile et al[10] as germinal matrix hemorrhage (IVH Grade I), or IVH Grade II, Grade III, or Grade IV based on review of standard coronal and sagittal images. IVH will be assessed in the right and left hemispheres and both results recorded, and the highest grade used as the study endpoint. Cerebellar hemorrhage will be assessed from mastoid views. Cystic periventricular leukomalacia will be assessed as radiolucent cysts in the periventricular white matter.

Primary Outcome measure

IVH of any grade on head ultrasound 7-10 days after birth

Note: Infants who die prior to having a 7-10 day HUS will be considered adverse outcomes for the primary endpoint. If a surviving infant does not have a HUS performed day 7-10 this will be considered a protocol deviation, and for the purpose of the primary outcome measure, the first HUS after 10 days of age will be used.

Primary Adverse Event Outcome Measures

Maternal

1) Post-partum hemorrhage in the first 24 hours after delivery (≥1000 ml estimated blood loss) (y/n)
2) Maternal RBC transfusion (y/n)
3) Post-partum delivery-related infection prior to hospital discharge (y/n)
4) Retained placenta (y/n)

Neonatal

1) NICU admission hypothermia (<36.5°C) (y/n)
2) Pneumothorax requiring intervention (y/n)

Secondary Outcome Measures

Neonatal

1) **Delivery Room**

- o Apgar scores <5 at 5 minutes (y/n)

2) **First 24 hours after birth:**
   - o Lowest hematocrit (median, min, max, 25th & 75th percentiles)
   - o Medication for low blood pressure (e.g., hydrocortisone or vasopressors) (y/n)
3) **First 10 days after birth**
   - o Number of RBC transfusions since birth (median, min, max, 25th & 75th percentiles)
4) **Hospital course until 36 weeks' PMA**
   - o Composite severe brain injury (y/n) based on local radiologist's read of the early or late HUS and defined as the presence of:
     - Grade 3-4 IVH or
     - Cerebellar hemorrhage or
     - Cystic periventricular leukomalacia (cPVL)
   - o Death prior to 36 weeks PMA (y/n)

Tertiary Outcome Measures

Neonatal

1) **Delivery Room**
   - o Apgar scores (1, 5, 10 minutes)
   - o Umbilical cord venous and arterial pH
   - o Intubation in delivery room
   - o Chest compressions or epinephrine
   - o Volume bolus given in delivery room
2) **First 24 hours after birth**
   - o NICU Admission temperature
   - o Red blood cell transfusion
   - o Highest hematocrit (before transfusion)
   - o Lowest mean arterial blood pressure
   - o Any volume bolus in the first 24 hours including in the delivery room, and including NICU packed red blood cells, platelets, plasma, saline, other fluid bolus
   - o SNAPPE-II score (Score for Neonatal Acute Physiology-Perinatal Extension) in the first 12 hours after birth
3) **First 10 days after birth**
   - o Surfactant administration
   - o Days on mechanical ventilation
   - o Highest bilirubin
   - o Days on phototherapy
4) **Hospital course until 36 weeks' PMA**

- IVH (most severe grade during hospitalization)
- Cerebellar hemorrhage
- Cystic periventricular leukomalacia (cPVL)
- Patent Ductus Arteriosus (PDA) requiring pharmacological or surgical treatment
- Early-onset septicemia (<72h from birth) (positive blood culture and at least 5 days of antibiotic therapy)
- Spontaneous intestinal perforation (SIP) requiring surgery or peritoneal drain
- Necrotizing enterocolitis (Modified Bell's stage 2-3)
- Bronchopulmonary dysplasia (receiving continuous supplemental oxygen at 36 weeks PMA)
- Severe ROP (stage 3 or treated with laser or bevacizumab) prior to 36 weeks PMA

Safety

Safety will be monitored in all randomized/on-study mothers and infants by comparing primary and secondary outcomes and reportable adverse and serious adverse events between study arms, as described in sections 8 and 9. This includes but is not limited to outcomes reported to be different in preterm infants undergoing delayed versus immediate cord clamping such as hematocrit in the first 24h after birth, bilirubin in the first 10d after birth, indicators of adverse cardiovascular transition in the delivery room and first 24h after birth, intraventricular hemorrhage, and other complications of extreme prematurity. The UVA Study Team and DSMC will regularly review these events and diagnoses for differences between study arms and differences from standard rates for EPT infants in international databases such as Vermont Oxford Network.

7 STATISTICAL CONSIDERATIONS

Study Design

The study is designed as a Phase III, randomized, multicenter comparative study of standard 30-60 seconds cord clamping with ventilatory assistance after cord clamping (Standard) versus 120 seconds cord clamping with ventilator assistance prior to cord clamping (VentFirst) in extremely preterm infants (EPT, 23 $^{0/7}$ – 28 $^{6/7}$ weeks' gestational age(GA)). Specifically, the study is designed to estimate the difference in survival without IVH on the first head ultrasound 7-10 days following birth between the two interventions in two cohorts of EPT infants.

two cohorts:	1) EPT infants **not breathing well** 20-30 seconds from delivery (apneic or gasping)<br>2) EPT infants **breathing well** 20-30 seconds from delivery (crying or breathing regularly)	
two stratification factors	1) Gestational age (2 levels):<br>GA 23 $^{0/7}$ – 25 $^{6/7}$ weeks; GA 26 $^{0/7}$ – 28 $^{6/7}$ weeks	2) Site
two interventions:	Arm 1) "Standard" (30-60 seconds cord clamping) with ventilatory assistance after cord clamping<br>Arm 2) "VentFirst" (120 seconds cord clamping with ventilatory assistance from 30-120 seconds)	

Accrual

We recognize that target accrual will be limited by two major factors:

1) Ability to obtain informed consent (some mothers will deliver before consent can be obtained, and others will decline the study)
2) Of the mothers giving informed consent, some will deliver before 23 or after 29 weeks and others will deliver very quickly before randomization and equipment set-up can be accomplished.

Taking into account these restrictions, we anticipate being able to enroll, randomize, and carry out the study protocol on ~ 35 to 40% of the estimated 2500 EPT deliveries over the study period. Target accrual is estimated at 940 eligible EPT infants, thus, accrual to the study should be completed within 4 ½ years of study initiation with yearly accrual rates of approximately 10%, 20%, 25%, 25% and 20% of the total.

Randomization and stratification

For this study, the randomization unit is the mother and the analysis unit is the EPT infant. Thus, twins are assigned the same intervention. Within

each site, mothers will be stratified by expectant infant GA categories without regard to cohort. Initial randomization will occur with equal allocation to each arm using a stratified block randomization scheme with varying block sizes (of size 2-6). Although we cannot determine cohort designation (breathing well or not) at randomization, given the large target sample size we assume that the randomization process will result in approximate balance between arms within cohort. However, if a significant imbalance is noted in cohort 1 as assessed by the DSMC then a weighted minimization scheme will be used to maintain arm balance within cohort 1. If balance is maintained, infants will continue to be randomized with equal allocation to each arm using the stratified block randomization scheme. Given that a 40/60% or a 30/70% arm imbalance reduces power to 78% or 73%, respectively, the randomization scheme will be modified if a 30/70% arm imbalance is observed in cohort 1, or as deemed appropriate by the DSMC. Note, the probability of a 30/70 split or more extreme in either arm in cohort 1 is <0.001.

Primary endpoint

The primary endpoint is based upon the central review evaluation and is the proportion of EPT infants who are alive without any grade IVH (including grade I germinal matrix hemorrhage) on head ultrasound obtained 7-10 days following birth. EPTs will be followed until 36 weeks post-menstrual age or discharge home, whichever comes first.

Primary hypothesis

For each cohort, the primary null hypothesis of the study is that there is no difference between the proportions of EPT infants who are alive without any grade IVH on head ultrasound obtained 7-10 days from birth for Standard versus VentFirst. The alternative hypothesis of the study is that EPT are less likely to have IVH on head ultrasound obtained 7-10 days from birth if they receive assisted ventilation from 30 to 120 seconds followed by cord clamping, compared to those with cords clamped at 30-60 seconds, followed by assisted ventilation.

$$H_o: p_{Standard} = p_{VentFirst}$$

$$H_a: p_{Standard} \neq p_{VentFirst}$$

Study Size Considerations

It is hypothesized that the investigational approach (VentFirst) improves outcomes in both cohorts with a greater effect in cohort 1 (not breathing

well), thus, the assumption of a common effect is violated and the two cohorts will be assessed separately. However, target sample size is based upon attaining a sufficient number of accruals to both cohorts. Based on discussions with investigators who have recently conducted delayed cord clamping trials in preterm infants,[11] in addition to experience in our own Pilot Trial, it is anticipated that ~50-65% of EPTs infants will be categorized as 'breathing well.'

The Cochrane meta-analysis of the effect of timing of umbilical cord clamping in preterm infants (delayed 30 or more seconds versus immediate) reported an estimated odds ratio (95% CI) of 0.59 (0.41-0.85) for survival without IVH. The Cochrane analysis included some infants up to 37 weeks gestational age where the differences in outcome affected by DCC may not be as large as might be expected in a cohort restricted to more immature infants. Furthermore, by restricting Cohort 1 to EPT infants who are not breathing well, it is hypothesized that the outcome effect will be even greater. Therefore, for this study we expect to see a larger reduction in our primary endpoint in the EPT infants in cohort 1(odds ratio of 0.5) and a reduction on the order of magnitude observed in the Cochrane analysis or slightly more (odds ratio of 0.59 or 0.55) in the EPT infants in cohort 2.[3]

Within each site, infants will be stratified by gestational age (GA 23 $^{0/7}$ to 25 $^{6/7}$ weeks; GA 26 $^{0/7}$ to 28 $^{6/7}$ weeks). Numbers of inborn infants 24-28 weeks' GA without major birth defects at Vermont Oxford Network (VON) centers in the United States (670 centers) born in 2013 were used as the baseline estimates of the primary event rate.[12] The information is summarized in the following table.

Strata: GA week/day	Proportion of newborns in each strata	"Success" % Alive without IVH at day 7-10	"Failure" 1-%Success
23 $^{0/7}$ to 25 $^{6/7}$	0.38	44.9%	55.1%
26 $^{0/7}$ to 28 $^{6/7}$	0.62	74.4%	25.6%
combined	1.00	63.0%	37.0%

We note that for sample size calculations we do not have numbers by site and thus assume a common effect, resulting in calculations that are based upon a randomized stratified design, which divides the sample among 2 strata at the assumed proportions observed in the VON data, assuming an uncorrected Cochran-Mantel-Haenszel test statistic with a two-sided 5% level test for *each* cohort, separately. Specifically, optimal target accrual for

the primary cohort of interest (cohort 1), summed across all strata, of 172 EPT infants in each intervention arm achieves approximately 80% power to test for a null odds ratio of 1.0 versus an alternative odds ratio of 0.5 (or a difference of 14% in rates). For cohort 2, optimal target accrual of 298 EPT infants in each intervention arm achieves approximately 80% power to test for a null odds ratio of 1.0 versus an alternative odds ratio of 0.55 (or a difference in rates of 12.5%). Shown in the table are estimated power calculations to assess each cohort assuming varying accrual proportions to the two cohorts and alternative odds ratios since accrual to each cohort cannot be controlled. The PASS 11 Power Analysis and Sample Size package was used to generate the sample size estimates.

For total target accrual of N=940 EPT infants ($N=N_1+N_2$)<br>Assuming equal allocation (1:1) to intervention arms within each breathing cohort						
Outcome	Sample size N_1, N_2	Proportion $N_1 : N_2$	Alternative odds ratio (difference)		Power	
					Cohort 1	Cohort 2
**Target***	**344, 596**	37%:63%	0.50	(14%)	**80%**	**96%**
			0.55	(12½%)	68%	**89%**
			0.59	(11%)	58%	**81%**
Possible	376, 564	40%:60%	0.50	(14%)	**83%**	**95%**
			0.55	(12½%)	72%	**88%**
			0.59	(11%)	62%	79%
Possible	470, 470	50%:50%	0.50	(14%)	**91%**	**91%**
			0.55	(12½%)	**81%**	**81%**
			0.59	(11%)	72%	**72%**
* target accruals & hypothesized odd ratio alternatives per cohort are noted in bold						

If we set total target accrual to 940 eligible EPT infants and assume that at least 37% of accruals fall into cohort 1 then we will have approximately 80% power to detect an odds ratio of 0.5 for VentFirst compared to Standard for cohort 1 and we will have approximately 81% power to detect an odds ratio of 0.59 for VentFirst compared to Standard for cohort 2. Power increases for cohort 1 assessment as the proportion of accruals to that cohort increases and decreases in cohort 2 to as low as 72% for an odds ratio of 0.6. However, increasing our ability to assess the primary hypothesis at the cost of reducing the secondary is reasonable and still provides sufficient power to detect an odds ratio of 0.55. Given these conditions we will require accrual of at least 344 EPT infants to Cohort 1. If our assumptions on accrual proportions are incorrect and more than 65% of EPT infants are being accrued to cohort 2 then accrual to cohort 2 will be halted once 600 EPT infants are accrued.

Interim Analysis and Stopping Guidelines

This study is designed using one interim look for efficacy of the primary outcome and one final look for a total of 2 planned analyses of the primary outcome in cohort 1. There are no interim analysis plans for cohort 2 or for futility; however, the proposed timeline can be modified based upon input from the DSMC. The guidelines serve as a trigger for consultation with the DSMC for additional review and are not formal "stopping rules" that would mandate automatic closure of study accrual. Adverse events and other safety endpoints will be monitored regularly and reported at each meeting of the DSMC.

Interim Analysis for Efficacy

The plan is for the interim analysis to be performed after approximately 2/3 of the EPT infants in cohort 1 have been assessed for the primary endpoint. The proposed stopping bounds used the error spending function method and were based upon accumulated information of 67% and 100%, and percent of alpha spent of 20% and 100% at the interim and final analyses, respectively. The bounds were generated under the assumptions specified for target sample size for cohort 1 with equal allocation and were based upon 50,000 simulations. The PASS 11 Power Analysis and Sample Size package was used to generate the significant boundaries. Group sequential trials with sample sizes of 172/arm with complete information at the final look achieve 80% power to detect an odds ratio of 0.5 of the VentFirst group proportion to a Standard group proportion of 0.37 at the 0.47 significance level (alpha) using a two-sided Mantel-Haenszel Z-Test.

Alpha spending and null hypothesis simulation results (50,000 simulations)								
Cohort (target)	Look	% Accumulated Information	Z-value scale	p-value scale	Target		Actual	
					Spending function alpha	Cum. spending function	Alpha spent	Cum. alpha spent
1	1st	67%	+/-2.611	0.009	0.008	0.008	0.008	0.008
	Final	100%	+/-1.987	0.047	0.042	0.050	0.041	0.049

Guidelines for Safety Monitoring

The DSMC will receive periodic safety reports of all adverse events and serious adverse events on the timeline specified in the DSMC charter. Monitoring of high grade (grade III-IV) IVH will be conducted monthly by the study PIs, and if rates approach near pre-set thresholds, the DSMC chair will be notified. The stopping guidelines serve as a trigger for

consultation with the DSMC for additional review. Statistical monitoring bounds for safety will be limited to the cumulative incidence of high grade IVH which will be monitored separately for each treatment arm within each cohort. Results from VON indicate high grade IVH event rate of 9.6%, 13.0% and 18.9% for the mean, 75th and 90th percentile, respectively. The upper boundary of a sequential probability ratio test (SPRT) based upon a binomial test of proportions of babies with high grade IVH will be used for monitoring to protect against excessive high grade rates. The stopping boundary are for a SPRT contrasting an acceptable rate of 9.6% versus a high rate of 18.9% of high grade IVH rate, with nominal type I and II errors of 5% and 20%, respectively. The slope of the parallel lines for monitoring is 0.1381 and the intercept is 3.5277. If a stopping bound is crossed then accrual to the study will be suspended until the DSMC can review the data, and determine if the study should continue, be amended or be closed to further accrual.

Demographic and Baseline Characteristics

Demographic and baseline characteristics will be summarized for all eligible mothers and EPT infants registered to the study by cohort and intervention arm.

Analysis Plan

Analysis Populations:

Intent-to-treat (ITT)

The primary analysis will be based on all randomized eligible EPT infants, and will be based on the arm they were randomized to regardless of intervention received (intent-to-treat).

Safety population

All randomized mothers and EPT infants will be evaluable for adverse events from the time of delivery.

Per Protocol population

A per protocol population will be used for secondary sensitivity analyses of the primary and secondary outcomes and will include all randomized EPT infants that do not have protocol deviations: 1) Eligibility violation 2) Intervention non-adherence 3) Intervention crossover 4) Missing primary outcome information.

Analyses of the Primary Endpoint

The primary outcome of the trial is based upon the central review and is the proportion of EPT infants who are alive without any grade IVH on head ultrasound obtained 7-10 days following birth. (Section 6.1.1) EPTs infants will be followed until 36 weeks PMA or discharge home or death, whichever comes first. The primary outcome will be assessed within each cohort separately. The primary outcome classification is binary and the stratified Cochran-Mantel-Haenszel test for relative risk will be used for the comparison of intervention arms stratified by gestation age (GA) at randomization. A 95% confidence interval for the relative risk within cohort will be constructed. Although the randomization unit is the mother, the analysis unit is the infant. Initial analyses will assume independence for twin outcomes. Subsequent analyses may employ a permutation test to confirm the results under the assumption of independence.

Analyses of the Secondary and Tertiary Endpoints

Adverse events will be tabulated by frequency and severity, overall and by arm as well as by cohort. Differences in the level of adverse events by study intervention will be assessed by classifying them as severe or not severe and examining the relative proportion of severe events.

Secondary endpoints for mothers and EPT infants are listed in Section 6.1.2. All endpoints will be summarized with descriptive measures by arm within cohort and overall. For all measures, initial analyses will be performed within cohort. In general, regression analysis consistent with the scale of the outcome measure will be used; logistic regression for binary outcomes; linear regression for continuous outcomes; Poisson or negative binomial for count outcomes all with independent variables for stratification factor and intervention assignment. 95% confidence intervals around the estimates will be constructed. The product-limit method of Kaplan-Meier will be used to estimate time to event type distributions such as survival to 36 weeks PMA and a stratified log rank test will be used to assess differences in survival by arm.

Handling protocol deviations or losses to follow-up

All randomized on-study EPT infants will be assessed for study endpoints even if there are major protocol deviations or they are deemed ineligible. For the primary outcome, any infant that dies at <7 days will be considered an intervention failure. For any surviving infant who has not had a HUS day 7-10 this will be a protocol deviation and there will be a reminder and an

extension to day 14. If a surviving infant has no HUS from day 7-14 this will be a protocol violation and the next available HUS after day 14 will be used for determination of the primary outcome. For secondary outcomes it is highly unlikely that data will be unavailable in the medical records of the delivery hospital until discharge home or 36 weeks PMA (whichever comes first). For infants who are transferred, study coordinators will contact the transfer hospital for secondary outcomes to 36 weeks PMA, with maternal consent. In the rare instances where there is insufficient information, secondary outcomes will be assessed based upon the latest information after consensus review by PIs or the DSMC, as described in the MOP.

Participant Enrollment and Follow-Up

As noted in Section 7.2, we anticipate being able to obtain consent, randomize, and perform the study intervention for about 40% of the estimated 2500 EPT births at all study centers over the 5 year period of the study, which will allow us to reach the targeted enrollment of 940 EPT infants.

8 ADVERSE EVENTS AND REPORTING

Extremely preterm infants commonly have multiple clinical complications and laboratory abnormalities associated with prematurity, and those listed in section 6will be collected and analyzed as secondary outcomes. These outcomes will be collected in the MuSIC database at times indicated in the MOP and reviewed monthly by the UVA Study Team. The DSMC will receive regular reports of secondary outcomes and compare rates in infants in the two study arms and compare overall rates in study infants to reported rates for EPT infants at NICUs in the Vermont Oxford Network or similar databases. If there is a significant imbalance of secondary outcome rates between the study arms or compared to current EPT rates, the proper course of action will be discussed by the study PIs and the DSMC.

Adverse events deemed related (definitely, probably or possibly) to a mother or infant being in the study will be collected and reported, as defined below. Recording of such adverse events will start at the time of the study intervention (birth) and will stop at the time of hospital discharge after delivery for mothers and at discharge home or 36 weeks PMA for infants. Any adverse events not resolved at the time a subject goes off study will be recorded as ‘ongoing’.

Adverse events must be assessed for expectedness, severity, attribution (relatedness to the study intervention), and seriousness by the site Investigator, or another qualified study team member.

Adverse events deemed related to the intervention must be reported as SAEs if they become serious.

Definitions

Adverse Event

Generally, an adverse event is any untoward medical occurrence in a subject who has received an investigational intervention.
Specific to this study, as noted above, common complications of prematurity will be collected and analyzed as secondary outcomes.
Adverse events judged to be related to being in the VentFirst study will be considered for AE reporting, as below.

Expectedness

The expectedness of the adverse event will be determined by the Investigator based on current literature and the Investigator's experience. We have included in this study adverse events that have been found to be related to the delayed cord clamping intervention in previous studies (see section 6). In addition, we have required the investigator to report any other unanticipated AEs that he/she feels may have been related to the intervention.

Severity

Where applicable, severity of adverse events can be described as indicated in the following table:

Table 2. Adverse Event Severity Grading Scale

Grade	Severity
1	Mild: transient symptoms, only requiring monitoring or symptomatic treatment and clinically significant
2	Moderate: illness or condition which requires new or significantly altered specific therapy
3	Severe: illness or condition unresponsive to medical therapy
4	Life-threatening: illness or condition complicated by acute life-threatening metabolic or cardiovascular complications (e.g., circulatory failure, hemorrhage, sepsis); life-threatening physiological consequences; or need for intensive or emergent invasive procedure (e.g., major surgery)
5	Death

Attribution Assessment (Relatedness)

The site principal investigator, or qualified designee, will evaluate all AEs and assess their relatedness, if any, to the study intervention. The following criteria will be used to define relatedness:

Related encompasses:
Definite: Direct association between AE and the study intervention
Probable: AE more likely explained by the study intervention
Possible: Study intervention and other cause could explain the AE equally well. (Can't rule either out)
Not related: AE clearly explained by another cause, requires other cause to be documented in the medical record.

Reportable Adverse Events

The adverse events listed below may be related to the subject being in the study and are the only adverse events that will require reporting.

**Adverse Event**	**Timing**
**Mother**	
Delivery-related infection	Prior to discharge
Retained placenta	
Other adverse events deemed by site PI to be definitely or probably related to being in the study	Any time
**Infant**	
Hypothermia <36°C on admission to NICU	NICU admission
Medication given for low blood pressure	1st 24 hours after birth
Anemia: hematocrit <30% (Hgb < 10 g/dl)	1st 24 hours after birth
Polycythemia: hematocrit >65% (Hgb > 22 g/dl)	1st 24 hours after birth
Bilirubin >12 mg/dl	1st 10 days after birth
IVH grade 1-2	prior to 36 weeks PMA
Cystic periventricular leukomalacia	prior to 36 weeks PMA
Necrotizing Enterocolitis (Modified Bell's stage 2-3)	prior to 36 weeks PMA
Other adverse events deemed by site PI to be definitely or probably related to being in the study	Any time

Reportable Serious Adverse Events

Generally, a serious adverse event is one that is temporally associated with the subject's participation in a research study that results in death or disability, is life-threatening, or prolongs hospitalization. Many of the typical complications of prematurity fit this description and only the following SAEs possibly related to the study will be reported as such.

Serious Adverse Event	Timing
**Mother**	
Any condition leading to death	Prior to discharge
Post-partum hemorrhage ≥ 1000 mL	In the first 24h after delivery (including delivery room blood loss)
Serious unexpected adverse event judged to be definitely or probably related to being in the study	Any time
**Infant**	
Any condition leading to death	Prior to 36 wks PMA
Chest compressions or epinephrine in DR	In the delivery room
IVH grade 3-4	On 7-10 day HUS or 36 week PMA HUS
Cerebellar hemorrhage	On 7-10 day HUS or 36 week PMA HUS
Pneumothorax requiring intervention	1st 24 hours after birth
Exchange transfusion for hyperbilirubinemia	1st 10 days after birth
Serious unexpected adverse event judged to be definitely or probably related to being in the study	Any time

Death

The event or condition that caused the death should be recorded as the adverse event with the outcome of death. If the cause of death is unknown and cannot be ascertained at the time of reporting, then the event should be reported as an **"unexplained death"**. If the cause of death later becomes available (e.g., after autopsy), "unexplained death" should be updated with the documented cause of death. See section 8.2.1 for further instruction on SAE reporting. Each study site will also report a study

death to its own IRB or IEC (Institutional Ethics Committee) per site institutional policy.

Unanticipated Serious Adverse Event

An unanticipated serious adverse event is an event that meets the criteria of serious AND is not expected per information in the protocol, study-referenced literature or in the informed consent.

Data Reporting Requirements

Reporting by Participating Sites

Event	Reported to	Timeframe
Reportable Adverse Events	MuSIC database	Within 30 days of event reporting
Serious Adverse Event: Initial Report	UVA Clinical Trials Office	Within 24 hours of knowledge of event
Serious Adverse Event: Interim Follow-Up Report	UVA Clinical Trials Office	As data are available
Serious Adverse Event: Final Follow-Up Report	UVA Clinical Trials Office	Within 15 days of event occurrence
Serious Adverse Event: All Reports	MuSIC database	Within 7 days of report submission to UVA CTO

Reporting to Participating Sites

The study overall PIs or designees are responsible for providing safety updates to all participating sites per the DSMC Charter guidelines and the MOP.

IRB Reporting Requirements

SAEs and serious unexpected adverse events must be submitted to the site IRB according to the participating site institutional policies.

9 DATA SAFETY MONITORING PLAN

Data Collection / CRF Completion

The Principal Investigators will provide continuous monitoring of subject safety in this trial with periodic reporting to the DSMC.

All data should be entered into the MuSIC database in accordance with the data entry instructions in the MOP. Adverse events should be entered into MuSIC as outlined in section 8 and the MOP. All study data should be entered into MuSIC per the timeframes noted in section 6 and the MOP.

Data and Safety Monitoring Committee

The Data and Safety Monitoring Committee will be composed of nationally-recognized experts in the clinical aspects of high risk pregnancies and complex management of extremely premature infants. There will be at least one statistician and one or more members with experience and knowledge in ethical aspects of care of EPT infants. The membership will have expertise in current clinical trials conduct and methodology

Meetings will be in-person or by teleconference, will be coordinated by the UVA CTO, and will be held at least annually and as needed if protocol-related or safety issues are identified. Any SAE listed in the table in section 8.1.6 requires expedited reporting and will be reported to the Chair of the DSMC within the timeframe indicated in DSMC Charter and/or the MOP.

10 STUDY MANAGEMENT

Institutional Review Board (IRB) Approval and Consent

It is expected that the each sites IRB will have the proper representation and function in accordance with federally mandated regulations. The IRB must approve the consent form and protocol.

In obtaining and documenting informed consent, the investigator should comply with the applicable regulatory requirement(s), and should adhere to all ICH E6 principles and Good Clinical Practice (GCP), to ethical principles that have their origin in the Declaration of Helsinki.

Before recruitment and enrollment onto this study, the mother will be given a full explanation of the study and will be given the opportunity to review the consent form. Once this essential information has been provided to the mother and the investigator is assured that the mother understands the implications of participating in the study, the mother will be asked to give consent to participate in the study by signing an IRB -approved consent form.

Prior to a mother's participation in the trial, the written informed consent form should be signed and personally dated by the subject and by the person who conducted the informed consent discussion.

Adherence to the Protocol

Except for an emergency situation in which proper care for the protection, safety, and well-being of the study subject requires alternative treatment, the study shall be conducted exactly as described in the approved protocol.

Emergency Modifications

Investigators may deviate from the protocol to eliminate an immediate hazard to subjects without prior IRB approval. For any such emergency, a protocol deviation or violation form should be submitted to the UVA CTO as soon as possible, and the UVA Study Team will decide whether IRB modifications are required and reported to all participating sites.

Other Protocol Deviations/Violations

Protocol Deviations: A protocol deviation is any unplanned variance from an IRB approved protocol that:

- Is generally noted or recognized after it occurs

- Has no substantive effect on the risks to research participants
- Has no substantive effect on the scientific integrity of the research plan or the value of the data collected
- Did not result from willful or knowing misconduct on the part of the investigator(s).

Study personnel will record the deviation, and report to any sponsor or data and safety monitoring committee in accordance with their policies. Deviations should be summarized and reported to the IRB at the time of continuing review.

Protocol Violations: An unplanned protocol variance is considered a violation if the variance:

- Has harmed or increased the risk of harm to one or more research participants.
- Has damaged the scientific integrity of the data collected for the study.
- Results from willful or knowing misconduct on the part of the investigator(s).
- Demonstrates serious or continuing noncompliance with federal regulations, State laws, or University policies.

Violations should be reported by study personnel to the IRB within one (1) week of the investigator becoming aware of the event.

Record Retention

Study documentation includes all Case Report Forms, data correction forms or queries, source documents, Sponsor-Investigator correspondence, monitoring logs/letters, and regulatory documents (e.g., protocol and amendments, IRB correspondence and approval, signed subject consent forms).

Source documents include all recordings of observations or notations of clinical activities and all reports and records necessary for the evaluation and reconstruction of the clinical research study.

Government agency regulations and directives require that all study documentation pertaining to the conduct of a clinical trial must be retained by the study investigator. Study documents should be kept on file until six years after the completion and final study report of this investigational study.

Obligations of Investigators

The Overall Principal Investigators are responsible for overseeing conduct of the trial and data collection, all applicable local regulatory laws and regulations and/or the Declaration of Helsinki. The Site Investigators are responsible for the conduct of the clinical trial at the site in accordance with Title 21 of the Code of Federal Regulations, The Site Investigator is responsible for personally overseeing the treatment of all study subjects. The Site Investigator must assure that all study site personnel, including sub-investigators and other study staff members, adhere to the study protocol and all GCP regulations and guidelines regarding clinical trials both during and after study completion. It is the responsibility of the Site Investigator to ensure that all study site personnel are aware that the study protocol and all data generated is confidential and should not be disclosed to third parties (with the exception of local and national regulatory bodies which require access for oversight purposes).

The Site Investigator at each institution or site will be responsible for assuring that all the required data will be collected and entered onto the Case Report Forms. Periodically, remote or on-site monitoring visits will be conducted and the Site Investigator must provide adequate source documentation to permit verification of proper entry of data.

11 REFERENCES

1. Institute of Medicine (US) Committee on Understanding Premature Birth and Assuring Healthy Outcomes; Behrman RE, Butler AS, editors. Preterm Birth: Causes, Consequences, and Prevention. Washington (DC): National Academies Press (US); 2007. 11, Neurodevelopmental, Health, and Family Outcomes for Infants Born Preterm. Available from: http://www.ncbi.nlm.nih.gov/books/NBK11356/

2. Volpe JJ. Intraventricular hemorrhage in the premature infant-current concepts. Ann Neurol. 1989Jan;25(1):3-11.

3. Rabe H, Diaz-Rossello JL, Duley L, Dowswell T. Effect of timing of umbilical cord clamping and other strategies to influence placental transfusion at preterm birth on maternal and infant outcomes. Cochrane Database of Systematic Reviews 2012

4. Polglase GR, Dawson J a., Kluckow M, et al. Ventilation Onset Prior to Umbilical Cord Clamping (Physiological-Based Cord Clamping) Improves Systemic and Cerebral Oxygenation in Preterm Lambs. PLoS One. 2015;10(2)

5. Posencheg M, Kirpalani H. Placental Transfusion at Birth: Do We HaveAll of the Answers? JAMA Pediatr. 2015 Jan;169(1):9-11

6. Hooper SB, te Pas AB, LangJ, van Vonderen JJ, Roehr CC, Kluckow M, Gill AW, WallaceEM, Polglase GR. Cardiovascular transition at birth: a physiological sequence. Ped Res. 2015 May, 77 (5), 608-614

7. Committee on Obstetric Practice. Committee Opinion: Timing of Umbilical Cord Clamping After Birth. Obstet Gynecol. 2012;120(6).

8. Wyckoff MH, Aziz K, Escobedo MB, Kapadia VS, Kattwinkel J, Perlman JM, Simon WM, Weiner GM, Zaichkin, JG. Part 13: neonatal resuscitation: 2015 American Heart Association Guidelines Update for Cardiopulmonary Resuscitation and Emergency Cardiovascular Care. Circulation. 2015;132(suppl 2):S543–S560

9. Perlman JM, Wyllie J, Kattwinkel J, Wyckoff MH, Aziz K, Guinsburg R, Kim HS, Liley HG, Mildenhall L, Simon WM, Szyld E, Tamura M, Velaphi S; on behalf of the Neonatal Resuscitation Chapter Collaborators. Part 7: neonatal resuscitation: 2015 International Consensus on Cardiopulmonary Resuscitation and Emergency Cardiovascular Care Science With Treatment Recommendations. Circulation. 2015;132(suppl 1):S204–S241

10. Papile L, Burstein J, Burstein R, Kofer H. Incidence and evaluation of subependyma l haemorrhage: a study of children with a birthweight less than 1500 g. J Pediatr 1978; 92: 529–

11. Gregory KE, Phillips M, Van Marter LJ. Necrotizing Enterocolitis in the Premature Infant. Adv Neonatal Care. 2011 Jun; 11(3): 155–166.)

12. Personal Communications, Jon Dorling, Nottingham, UK, Anup Katheria, San Diego, CA, Martin Kluckow, Sydney, AU

13. Data kindly provided by Drs. Jeffrey Horbar and Erika Edwards, Vermont Oxford Network, Burlington, VT.

14. Katheria A, Poeltler D, Durham J, Steen J, Rich W, Arnell K, Maldonado M, Cousins L, Finer N. Neonatal Resuscitation with an Intact Cord: A Randomized Clinical Trial. J. Pediatr. 2016 Nov: 178:75-80e3. doi: 10.2016/j.peds.2016.07.053. Epub 2016 Aug 26.

15. Postpartum hemorrhage. Practice Bulletin No. 183. American College of Obstetricians and Gynecologists. Obstet Gynecol 2017;130: e168–86.

16. Quantitative blood loss in obstetric hemorrhage. ACOG Committee Opinion No. 794. American College of Obstetricians and Gynecologists. Obstet Gynecol 2019; 134:e150-6.
