# Supplementary material for: Physiologic Transition During Delayed Cord Clamping With Assisted Ventilation in Preterm Infants: A Secondary Analysis of the VentFirst Trial
Source: JAMA Netw Open. 2025 Nov 24;8(11):e2545258. doi: 10.1001/jamanetworkopen.2025.45258 (PMC12645335; doi:10.1001/jamanetworkopen.2025.45258)
Supplement: Supplement 2. — eFigure 1. CONSORT Diagram of VentFirst Participants eTable 1. Primary Outcome and Delivery Room Measures With Potential Association Within Gestational Age Strata at Randomization eTable 2. Model Statistics eTable 3. Additional Infant Outcome Characteristics Used in Logistic Regression Models eFigure 2. Individual Infant Temperatures in the Delivery Room by Birth Weight, Mode Of Delivery (Vaginal Versus Cesarean Section), Cohort (Breathing Well Versus Not Breathing Well 30 seconds after birth), and Arm (Intervention Versus Control) [file jamanetwopen-e2545258-s002.pdf]

Supplemental Online Content

Fang JL, Fairchild KD, Petroni GR, et al. Physiologic transition during delayed cord clamping with assisted ventilation in preterm infants. *JAMA Netw. Open*. 2025;8(11):e2545258. doi:10.1001/jamanetworkopen.2025.45258

eFigure 1. CONSORT Diagram of VentFirst Participants

eTable 1. Primary Outcome and Delivery Room Measures With Potential Association Within Gestational Age Strata at Randomization

eTable 2. Model Statistics

eTable 3. Additional Infant Outcome Characteristics Used in Logistic Regression Models

eFigure 2. Individual Infant Temperatures in the Delivery Room by Birth Weight, Mode of Delivery (Vaginal Versus Cesarean Section), Cohort (Breathing Well Versus Not Breathing Well 30 Seconds After Birth), and Arm (Intervention Versus Control)

This supplemental material has been provided by the authors to give readers additional information about their work.

© 2025 Fang JL et al. *JAMA Network Open*.

eFigure 1. CONSORT diagram of VentFirst participants.

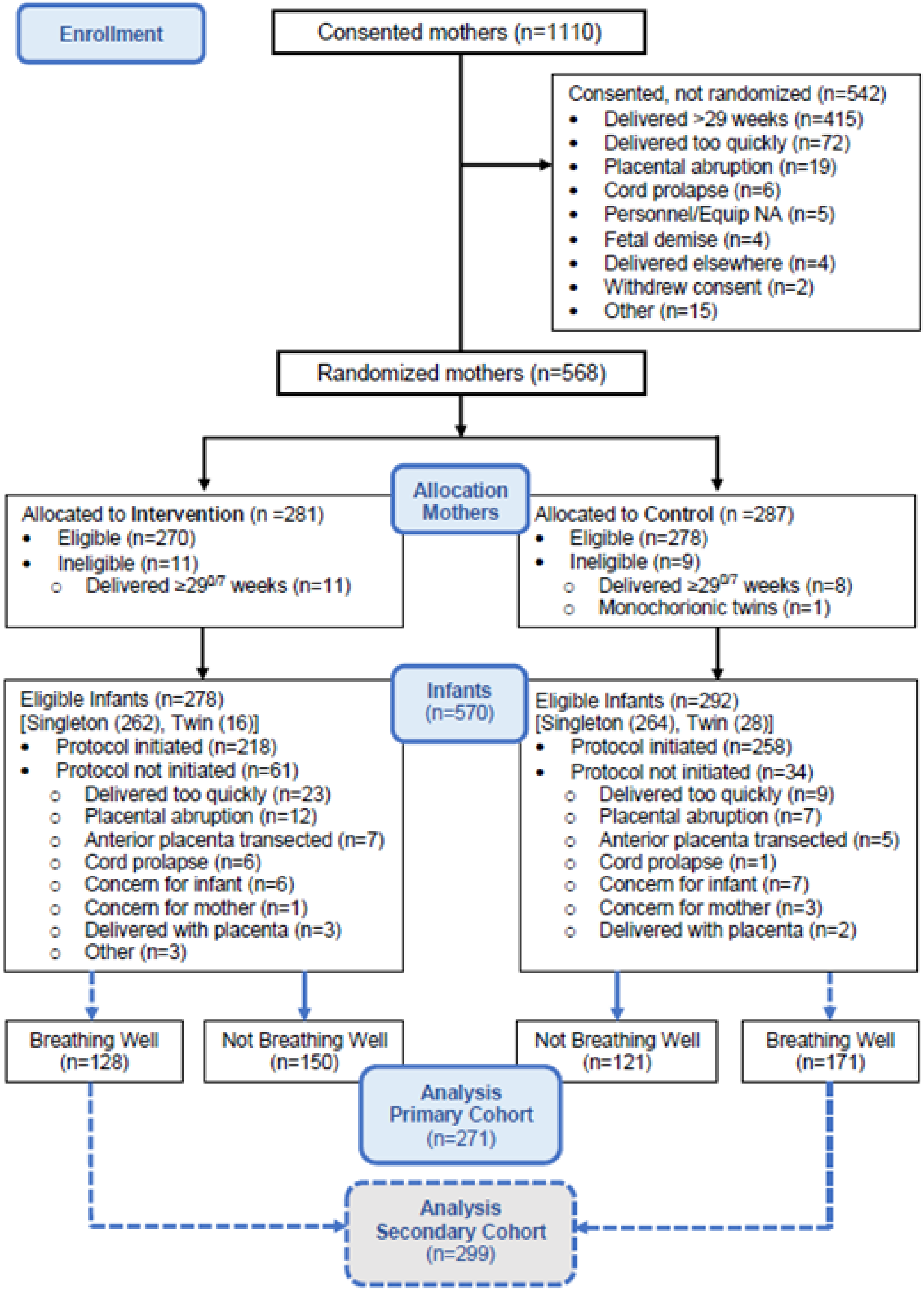

© 2025 Fang JL et al. *JAMA Network Open*.

eTable 1. Primary outcome and delivery room measures with potential association within gestational age strata at randomization.

	**Not breathing well at 30 sec**							
	GA 23 0/7 to 25 6/7				***GA 26 0/7 to 28 6/7***			
	**Intervention, No. (%) (n=71)**	**Control, No. (%) (n=61)**	**OR (95% CI)**	**MD (95% CI)**	**Intervention, No. (%) (n=79)**	**Control, No. (%) (n=60)**	**OR (95% CI)**	**MD (95% CI)**
**Primary outcome in the Delivery Room**								
Intubation (all)	53 (74.7)	46 (75.4)	0.96 (0.44 to 2.12)	NA	**18 (22.8)**	**29 (48.3)**	**0.32 (0.15 to 0.65)**	NA
**Other measures in the Delivery Room**								
Apgar at 1 minute, median (IQR)	**3 (1, 4)**	**2 (1, 3)**	NA	**0.83 (0.29 to 1.38)**	**4 (2, 5)**	**2.5 (1, 4)**	NA	**1.12 (0.48 to 1.76)**
Heart rate >100 bpm at 1 minute after birth, n (%)	**28 (39.4)**	**11 (18.0)**	**2.96 (1.32 to 6.64)**	NA	35 (44.3)	18 (30.0)	1.86 (0.91 to 3.77)	NA
Infant temperature <36.5°C, n (%) [a]	**29 (41.4)**	**14 (23.3)**	**2.32 (1.08 to 4.99)**	NA	22 (27.9)	11 (18.3)	1.72 (0.76 to 3.90)	NA
Infant temperature >37.5°C n (%) [a]	5 (7.1)	5 (8.3)	0.85 (0.23 to 3.08)	NA	7 (8.9)	6 (10.0)	0.88 (0.28 to 2.75)	NA

Abbreviations: GA, gestational age; IQR, interquartile range; OR, odds ratio; MD, mean difference; CI, confidence interval.

[a] = 3 infants (0.5%) had no recorded temperature; 38 (6.5%) infant temperatures in the DR were not available, NICU admission temperature was used.

	**Breathing well at 30 sec**							
	GA 23 0/7 to 25 6/7				***GA 26 0/7 to 28 6/7***			
	**Intervention, No. (%) (n=45)**	**Control, No. (%) (n=54)**	**OR (95% CI)**	**MD (95% CI)**	**Intervention, No. (%) (n=83)**	**Control, No. (%) (n=117)**	**OR (95% CI)**	**MD (95% CI)**
**Primary outcome in the Delivery Room**								
Intubation (all)	20 (44.4)	30 (55.6)	0.64 (0.29 to 1.42)	NA	8 (9.6)	16 (13.7)	0.67 (0.27 to 1.66)	NA
**Other measures in the Delivery Room**								
Apgar at 1 minute, median (IQR)	5 (4, 7)	6 (4, 7)	NA	0.01 (-0.65 to 0.68)	6 (5, 7)	6 (5, 7)	NA	0.02 (-0.49 to 0.53)
Heart rate >100 bpm at 1 minute after birth, n (%)	33 (73.3)	39 (72.2)	1.06 (0.44 to 2.57)	NA	67 (80.7)	93 (79.5)	1.08 (0.53 to 2.19)	NA
Infant temperature <36.5°C, n (%) [a]	17 (37.8)	11 (20.4)	2.37 (0.97 to 5.81)	NA	**14 (16.9)**	**37 (31.9)**	**0.43 (0.22 to 0.87)**	NA
Infant temperature >37.5°C n (%) [a]	**1 (2.2)**	**10 (18.5)**	**0.10 (0.01 to 0.82)**	NA	5 (6.0)	12 (10.3)	0.56 (0.19 to 1.64)	NA

Abbreviations: GA, gestational age; IQR, interquartile range; OR, odds ratio; MD, mean difference; CI, confidence interval.

[a] = 3 infants (0.5%) had no recorded temperature; 38 (6.5%) infant temperatures in the DR were not available, NICU admission temperature was used.

© 2025 Fang JL et al. *JAMA Network Open*.

eTable 2. Model statistics.

Model Outcome of Interest	N	n (%) with event	Likelihood ratio test Pr>ChiSq	Hosmer and Lemeshow Goodness-of-Fit Pr>ChiSq	C-statistic	Somers' D
Intubation	569	219 (38)	<0.001	0.620	0.87	0.74
Hypothermia	566	154 (27)	<0.001	0.740	0.75	0.50
Hyperthermia	566	51 (9)	0.043	0.220	0.72	0.45

© 2025 Fang JL et al. *JAMA Network Open*.

eTable 3. Additional infant characteristics used in logistic regression models.

**Outcome**	**Not breathing well at 30 sec (n=271)**	**Breathing well at 30 sec (n=299)**	**Total (n=570)**
GA at birth (wk), median (IQR)	26.0 (24.7, 27.4)	27.0 (25.7, 28.0)	26.6 (25.1, 27.8)
Categories, n (%)			
23 0/7 to 25 6/7	127 (46.9)	87 (29.1)	214 (37.5)
26 0/7 to 28 6/7	144 (53.1)	212 (70.9)	356 (62.5)
Time of cord clamping (sec), median (IQR)	63 (31, 120)	35 (10, 110)	66 (60, 120)
Cord clamping time categories, n (%) [a]			
<15 sec	74 (27.4)	16 (5.4)	90 (15.8)
15-29 sec	24 (8.9)	2 (0.7)	26 (4.6)
30-59 sec	62 (23.0)	15 (5.0)	77 (13.5)
60-119 sec	47 (17.4)	171 (57.2)	218 (38.3)
≥120 sec	63 (23.3)	95 (31.8)	158 (27.8)

Abbreviations: GA, gestational age; IQR, interquartile range.

[a] One NBW infant missing time of cord clamping

© 2025 Fang JL et al. *JAMA Network Open*.

eFigure 2. Individual infant temperatures in the delivery room by birth weight, mode of delivery (vaginal versus Cesarean section), cohort (breathing well versus not breathing well 30 seconds after birth), and arm (intervention versus control).

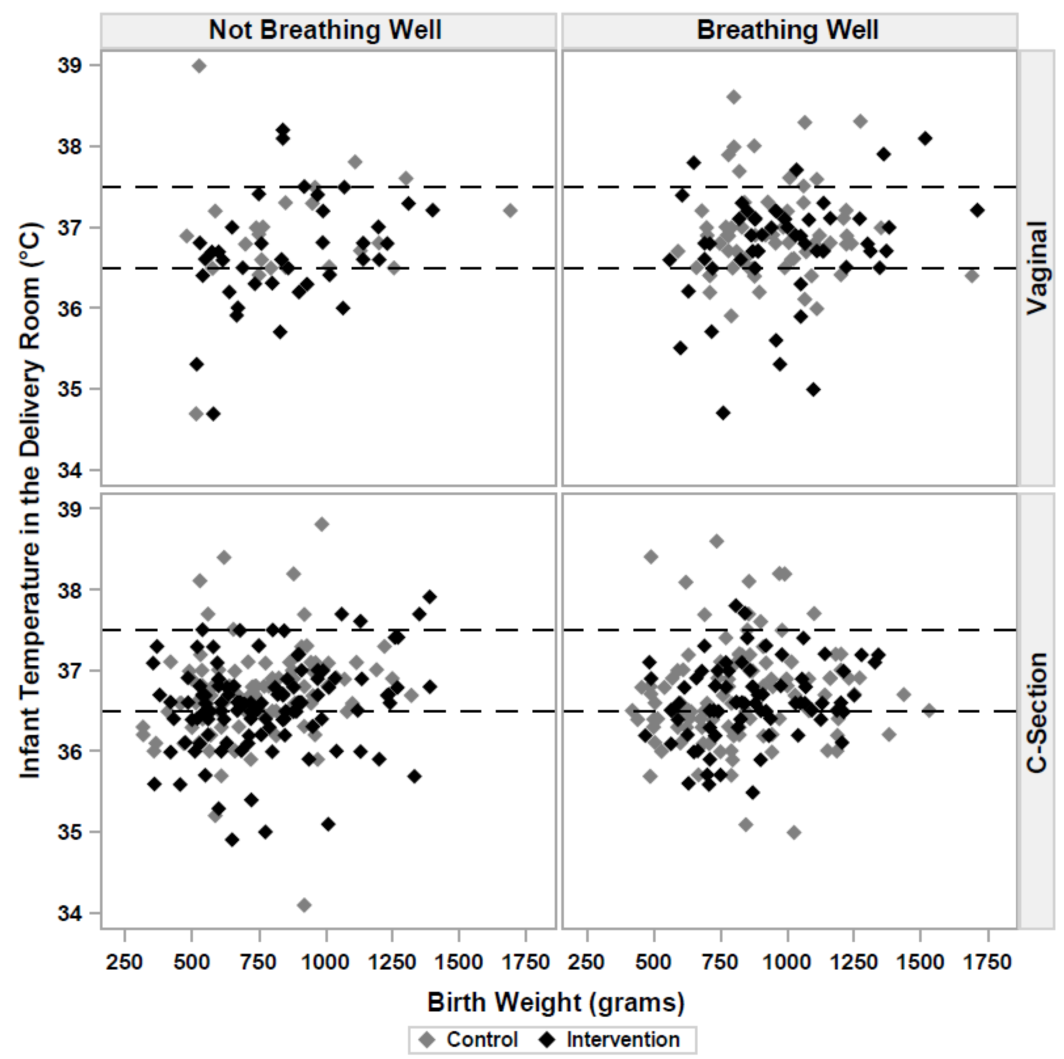

© 2025 Fang JL et al. *JAMA Network Open*.
