# Supplementary material for: Physiologic Transition During Delayed Cord Clamping With Assisted Ventilation in Preterm Infants: A Secondary Analysis of the VentFirst Trial
Source: JAMA Netw Open. 2025 Nov 24;8(11):e2545258. doi: 10.1001/jamanetworkopen.2025.45258 (PMC12645335; doi:10.1001/jamanetworkopen.2025.45258)
Supplement: Supplement 3. — Nonauthor Collaborators [file jamanetwopen-e2545258-s003.pdf]

Supplemental Online Content: Nonauthor Collaborators
*First name, last name, and suffix (if applicable) are required and will appear in PubMed.

*Group Name(s): The VentFirst Consortium							
***First Name and Middle Initial(s)**	***Last Name**	***Suffix (eg, Jr, III)**	Academic Degrees	Institution	Location (city, state/province, country)	Role or Contribution, eg, chair, principal investigator	Group (if more than 1 Group listed in the byline) and/or Subgroup (eg, Steering Committee)
Karen D	Fairchild		MD	University of Virginia School of Medicine	Charlottesville, VA, USA		
John	Kattwinkel		MD	University of Virginia School of Medicine	Charlottesville, VA, USA		
Gina M	Duda		BS	University of Virginia School of Medicine	Charlottesville, VA, USA		
Gina R	Petroni		PhD	University of Virginia School of Medicine	Charlottesville, VA, USA		
Nikole E	Varhegyi		MS	University of Virginia School of Medicine	Charlottesville, VA, USA		
Monika	Thielen		ART	University of Virginia School of Medicine	Charlottesville, VA, USA		
Christian A	Chisholm		MD	University of Virginia School of Medicine	Charlottesville, VA, USA		
Jennifer L	Fang		MD	Mayo Clinic College of Medicine and Science	Rochester, MN, USA		
Lavonne M	Liedl		RRT	Mayo Clinic College of Medicine and Science	Rochester, MN, USA		
Amy L	Amsbaugh		RRT	Mayo Clinic College of Medicine and Science	Rochester, MN, USA		
Susan	Niermeyer		MD	University of Colorado School of Medicine	Aurora, CO, US		
James A	Barry		MD	University of Colorado School of Medicine	Aurora, CO, US		
Amy	Lamprecht		BSN	University of Colorado School of Medicine	Aurora, CO, US		
Carolyn S	Berlinski		BSN	University of Colorado School of Medicine	Aurora, CO, US		

Supplemental Online Content: Nonauthor Collaborators
*First name, last name, and suffix (if applicable) are required and will appear in PubMed.

***First Name and Middle Initial(s)**	***Last Name**	***Suffix (eg, Jr, III)**	Academic Degrees	Institution	Location (city, state/province, country)	Role or Contribution, eg, chair, principal investigator	Group (if more than 1 Group listed in the byline) and/or Subgroup (eg, Steering Committee)
Jamie B	Warren		MD	Oregon Health & Science University	Portland, OR, USA		
Monica	Rincon		MD	Oregon Health & Science University	Portland, OR, USA		
Marya L	Strand		MD	Saint Louis University School of Medicine	St. Louis, MO, USA		
Justin B	Josephsen		MD	Saint Louis University School of Medicine	St. Louis, MO, USA		
Melissa	Hawkins		RN	Saint Louis University School of Medicine	St. Louis, MO, USA		
Sumesh P	Thomas		MBBS	University of Calgary	Calgary, AB, Canada		
Jacque-Lynne F	Johnson		PhD	University of Calgary	Calgary, AB, Canada		
Leigh	Irvine		MN	University of Calgary	Calgary, AB, Canada		
Anna-Maria	Ciorogariu-Ivan		BSc	University of Calgary	Calgary, AB, Canada		
Terri E	Gorman		MD	Brigham and Women's Hospital	Boston, MA, USA		
Bobbi J	Byrne		MD	Indiana University School of Medicine	Indianapolis, IN, USA		
Mark A	Underwood		MD	University of California, Davis School of Medicine	Sacramento, CA, USA		
Francis R	Poulain		MD	University of California, Davis School of Medicine	Sacramento, CA, USA		
Rosa R	Pesavento		BA	University of California, Davis School of Medicine	Sacramento, CA, USA		
Tina A	Leone		MD	Columbia University Medical Center	New York, NY, USA		
Brenda H	Law		MD	University of Alberta, Edmonton	Edmonton, AB, Canada		
Caroline	Fray		BSN	University of Alberta, Edmonton	Edmonton, AB, Canada		
Melba	Athaide		BSN	University of Alberta, Edmonton	Edmonton, AB, Canada		
Andrea F	Kane		MD	University of Alabama at Birmingham	Birmingham, AL, USA		

Supplemental Online Content: Nonauthor Collaborators
*First name, last name, and suffix (if applicable) are required and will appear in PubMed.

***First Name and Middle Initial(s)**	***Last Name**	***Suffix (eg, Jr, III)**	Academic Degrees	Institution	Location (city, state/province, country)	Role or Contribution, eg, chair, principal investigator	Group (if more than 1 Group listed in the byline) and/or Subgroup (eg, Steering Committee)
Colm P	Travers		MD	University of Alabama at Birmingham	Birmingham, AL, USA		
Waldemar A	Carlo		MD	University of Alabama at Birmingham	Birmingham, AL, USA		
Dorothy I	Bulas		MD	Children's National Medical Center	Washington D.C., USA		
Beth M	Kline-Fath		MD	Cincinnati Children's Hospital Medical Center	Cincinnati, OH, USA		
Monica	Epelman		MD	Nemours Children's Hospital	Orlando, FL, USA		
