# Supplementary material for: Physiologic Transition During Delayed Cord Clamping With Assisted Ventilation in Preterm Infants: A Secondary Analysis of the VentFirst Trial
Source: JAMA Netw Open. 2025 Nov 24;8(11):e2545258. doi: 10.1001/jamanetworkopen.2025.45258 (PMC12645335; doi:10.1001/jamanetworkopen.2025.45258)
Supplement: Supplement 4. — Data Sharing Statement [file jamanetwopen-e2545258-s004.pdf]

Data Sharing Statement

Fang. Physiologic Transition During Delayed Cord Clamping With Assisted Ventilation in Preterm Infants. *JAMA Netw Open*. Published November 24, 2025. doi:10.1001/jamanetworkopen.2025.45258

Data
Additional Information: Clinicaltrials.gov, NCT02742454 https://clinicaltrials.gov/study/NCT02742454?term=ventfirst&rank=2
Data available: No

Additional Information
Explanation for why data not available: Data sharing statement was included with the primary paper.
https://cdn.jamanetwork.com/ama/content_public/journal/jamanetworkopen/939364/zoi240401supp4_prod_1715361697.28687.pdf?
Expires=1748039580&Signature=VWqAibZUU8GqM8m2x15LmvB6wQ4tqNU8gKANzOGQGyw-d-
4GqMgX3Ohmt76sg40uEx1bKnv0USmz9aExujNcYaFhNgjVo4kmKxVAH9hvO7XkvEUN4~leXoVRO9BltrZp7J0cVd0F8p-
vsUiXZUuZfQOY8Kx3X0mHaOyIrQOXnxuAqKPdXDHnrTSslCZZFCpZUbY1C8O8vswSFJdi9AXpsZmAaN9a4cN~NssLQPRodaWD5mxwQkUFJ45zT~QW
Px2zKD26vKG2Nxn7sCd7nkGxYr~GL1ZdUnSayRCsmHZ77SxvY08rsgQ__&Key-Pair-Id=APKAIE5G5CRDK6RD3PGA
